# A scoping review of foot‐and‐mouth disease risk, based on spatial and spatio‐temporal analysis of outbreaks in endemic settings

**DOI:** 10.1111/tbed.14769

**Published:** 2022-12-13

**Authors:** Lina González Gordon, Thibaud Porphyre, Dennis Muhanguzi, Adrian Muwonge, Lisa Boden, Barend M. de C Bronsvoort

**Affiliations:** ^1^ The Epidemiology, Economics and Risk Assessment (EERA) Group, The Roslin Institute at The Royal (Dick) School of Veterinary Studies University of Edinburgh Easter Bush Midlothian UK; ^2^ Global Academy of Agriculture and Food Systems University of Edinburgh Easter Bush Midlothian UK; ^3^ Laboratoire de Biométrie et Biologie Evolutive Université de Lyon, Université Lyon 1, CNRS, VetAgro Sup Marcy‐l’Étoile France; ^4^ Department of Bio‐Molecular Resources and Bio‐Laboratory Sciences, College of Veterinary Medicine, Animal Resources and Biosecurity Makerere University Kampala Uganda

**Keywords:** Foot‐and‐Mouth Disease, Disease Outbreaks, Spatio‐Temporal Analysis, Risk Factors, Endemic Diseases

## Abstract

Foot‐and‐mouth disease (FMD) is one of the most important transboundary animal diseases affecting livestock and wildlife species worldwide. Sustained viral circulation, as evidenced by serological surveys and the recurrence of outbreaks, suggests endemic transmission cycles in some parts of Africa, Asia and the Middle East. This is the result of a complex process in which multiple serotypes, multi‐host interactions and numerous socio‐epidemiological factors converge to facilitate disease introduction, survival and spread. Spatial and spatio‐temporal analyses have been increasingly used to explore the burden of the disease by identifying high‐risk areas, analysing temporal trends and exploring the factors that contribute to the outbreaks. We systematically retrieved spatial and spatial‐temporal studies on FMD outbreaks to summarize variations on their methodological approaches and identify the epidemiological factors associated with the outbreaks in endemic contexts. Fifty‐one studies were included in the final review. A high proportion of papers described and visualized the outbreaks (72.5%) and 49.0% used one or more approaches to study their spatial, temporal and spatio‐temporal aggregation. The epidemiological aspects commonly linked to FMD risk are broadly categorizable into themes such as (a) animal demographics and interactions, (b) spatial accessibility, (c) trade, (d) socio‐economic and (e) environmental factors. The consistency of these themes across studies underlines the different pathways in which the virus is sustained in endemic areas, with the potential to exploit them to design tailored evidence based‐control programmes for the local needs. There was limited data linking the socio‐economics of communities and modelled FMD outbreaks, leaving a gap in the current knowledge. A thorough analysis of FMD outbreaks requires a systemic view as multiple epidemiological factors contribute to viral circulation and may improve the accuracy of disease mapping. Future studies should explore the links between socio‐economic and epidemiological factors as a foundation for translating the identified opportunities into interventions to improve the outcomes of FMD surveillance and control initiatives in endemic contexts.

## 1 INTRODUCTION

Foot‐and‐mouth disease (FMD), caused by the FMD virus (FMDv; genus *Aphthovirus*, family Picornaviridae), is a highly contagious viral disease circulating within cloven‐hoofed animal populations across the world (Rowlands, 2008). Its widespread distribution, transboundary nature and severe economic implications make FMD one of the most important livestock diseases globally (Knight‐Jones & Rushton, 2013). Clinically, it is characterized by the formation of vesicles in the tongue, hard palate, dental pad, lips, gums, muzzle, coronary band and interdigital space, accompanied by salivation, anorexia, depression and lameness, leading to poor production performance and low efficiency in livestock systems (Kitching, 2002a, 2002b, 2002c; Knight‐Jones & Rushton, 2013).

Although FMD is considered controlled in many high and middle‐income settings (World Organization for Animal Health Territories [WOAH] ‘Disease free territories’ with or without vaccination), the disease is still estimated to affect ∼77% of the livestock population around the world, particularly in Tropical Africa and Asia where it is considered endemic (World Organization for Animal Health, 2022a). In some of these countries, FMD outbreaks commonly recur despite the implementation of prevention and control strategies which include a combination of stamping‐out policies, pre‐emptive or emergency vaccination, movement restrictions, increased biosecurity, strengthened surveillance and community sensitization and education programmes (Blacksell et al., 2019; Maree et al., 2014). These strategies are implemented and enforced heterogeneously across countries due to differences in animal health priorities, and varying budget and logistical capabilities, and hence have resulted in partial or no success.

Multiple epidemiological factors converge to facilitate FMDv transmission, highlighting the complexity that underlies the mechanisms leading to viral introduction, spread and persistence in endemic settings (OIE, 2018; Santos et al., 2017; Squarzoni‐Diaw et al., 2021). A better understanding of the key factors associated with viral endemicity would support FMD control and prevention programmes particularly by identifying priority areas for targeted resource allocation and by supporting the design of robust policy alternatives to effectively tackle the disease and mitigate its impact.

Obtaining data for an accurate epidemiological picture of FMDv in endemic settings can be challenging. However, questions looking at the mechanisms and pathways by which FMDv remains endemic in some areas can be approached using outbreak records, a common and accessible data source to study infectious diseases. Countries often keep FMD outbreak data registries because outbreak detection and response are one of the key roles of veterinary services of government agencies, especially if the disease is of high economic importance or if it has been targeted for control and eventual eradication. FMD is one of the 85 WOAH‐listed diseases detailed in the animal terrestrial code, with official outbreak records collated and publicly available at the WOAH‐WAHIS database (World Organization for Animal Health, 2022c). This data catalogue is assembled on the basis of the reports compiled by local veterinary services across the world. Therefore, outbreak data is an essential feed for evidence‐based strategies to manage FMDv circulation such as the Progressive Control Pathway for Foot and Mouth Disease (PCP‐FMD) (FAO et al., 2018), an FAO‐led staggered control approach for FMD with a worldwide reach. Large‐scale outbreak data is central to understand the epidemiological situation, develop interventions and evaluate the progress of FMDv management approaches.

Outbreak data can be analysed using spatial and spatio‐temporal tools to explore the patterns and risk factors for FMD and for the identification of disease hotspots. These analyses are a major source for data‐driven decision‐making for local veterinary services in endemic settings. However, there are many tools used to test several spatio‐temporal hypotheses and achieve different objectives; this often results in outputs that cannot be compared directly without contextual awareness and sufficient background on the analytical tools used (Carpenter, 2001; Kanankege et al., 2020). For example, many studies examine the role of demographic, environmental, socio‐economic, landscape and other local features in influencing spatial and temporal variations in disease circulation, identification, or reporting. Critically, model parametrizations tend to be influenced by the postulated disease pathway linking the epidemiological factors and the outbreaks (Kraemer et al., 2019). It is this aspect that contextualizes the disease dynamics to a specific location. Therefore, the current literature on these tools in endemic settings is a collection of context‐specific insights, hence the need to review this body of work to gain insight into the global utility of these tools in the control of FMD in such settings.

Using a non‐systematic narrative review, Premashthira et al. (2011) documented the use of epidemiological simulation modelling and spatial analysis in disease control to understand FMD circulation. More recently, Souley Kouato et al. (2018) retrieved quantitative and qualitative FMD risk assessments published in endemic and non‐endemic settings across the world and critically analysed them from the perspectives of their implications for FMD prevention in Africa. Although these reviews have addressed some aspects related to FMD spatio‐temporal risk and summarize the results of several statistical models used to forecast FMD, it is still unclear which approaches have been selected to process FMD outbreak data collected in endemic settings and what are their findings when analysed in the light of the whole body of evidence.

The aim of this scoping review was to (a) systematically retrieve spatial and spatial‐temporal studies analysing FMDv distribution and patterns based on outbreak reports in endemic countries, (b) summarize key aspects of their methodological approach and (c) identify population‐level epidemiological factors used to map and understand FMDv risk in endemic contexts. The information collected through this synthesis process will ultimately serve to produce an evidence map of the current scientific literature and identify knowledge gaps and limitations of the studies. Moreover, the findings from this review could inform the standardization of modelling inputs and strategies for an improved comparability between their outputs.

## 2 METHODS

### 2.1 Methodological framework and review questions

We followed the methodological and reporting quality guidelines proposed by PRISMA‐ScR, an extension of the Preferred Reporting Items for Systematic Reviews – PRISMA – designed for Scoping Reviews for conducting and reporting all aspects of this literature synthesis (Tricco et al., 2018) (Table S1). A scoping review is a type of exploratory evidence synthesis designed to identify, summarize, map and highlight the knowledge and the potential gaps in a research area using a systematic, transparent, reproducible approach (Peters et al., 2015; Tricco et al., 2018). The questions addressed by this review were defined as follows: (a) Which analytical approaches are used to study geolocated (point) or areal (aggregated) FMD outbreak data in endemic settings? (b) What is known from the literature in relation to FMD risk based on the analysis of these outbreaks (e.g. identification of hotspots, clusters, seasonal or temporal trends)? (c) Which risk factors have been examined and which have been linked to the occurrence of outbreaks? and (d) What are the knowledge gaps based on the current scientific literature?

### 2.2 Eligibility criteria

This review included FMD studies incorporating spatial, temporal or spatio‐temporal approaches for data analysis and inference. Based on the classification proposed by Kanankege et al. (2020), studies can use different spatio‐temporal analytical tools for epidemiological research. These can be classified based on the purpose of the analysis on (a) visualization and description, (b) spatial or spatio‐temporal dependence and pattern recognition, (c) spatial smoothing and interpolation and (d) modelling and regression studies. Within the category of modelling and regression, we were particularly interested in studies designed to investigate epidemiological factors linked to FMD outbreaks at the population level or that integrated presumed FMD risk factors to project disease occurrence.

Thus, studies considered for inclusion were those that (a) reported FMD outbreak distribution/patterns or modelled FMD risk using geographically or/and temporally linked data; (b) used population outbreak data collected in FMDv endemic countries; (c) were published in a peer‐reviewed scientific journal between January 2000 and July 2022 in English or Spanish; and (d) documented FMD outbreak occurrence in livestock (e.g. cattle, small ruminants, pigs, camels and water buffalo). Studies that used data collected from farm outbreak surveys with self‐reported FMD occurrence and risk factors or that described outbreaks in wildlife species were excluded. Also, studies that were designed as outbreak investigations, risk assessments and narrative literature reviews documenting FMD occurrence and trends (without further data analysis) were out of the scope of this synthesis.

**FIGURE 1:**
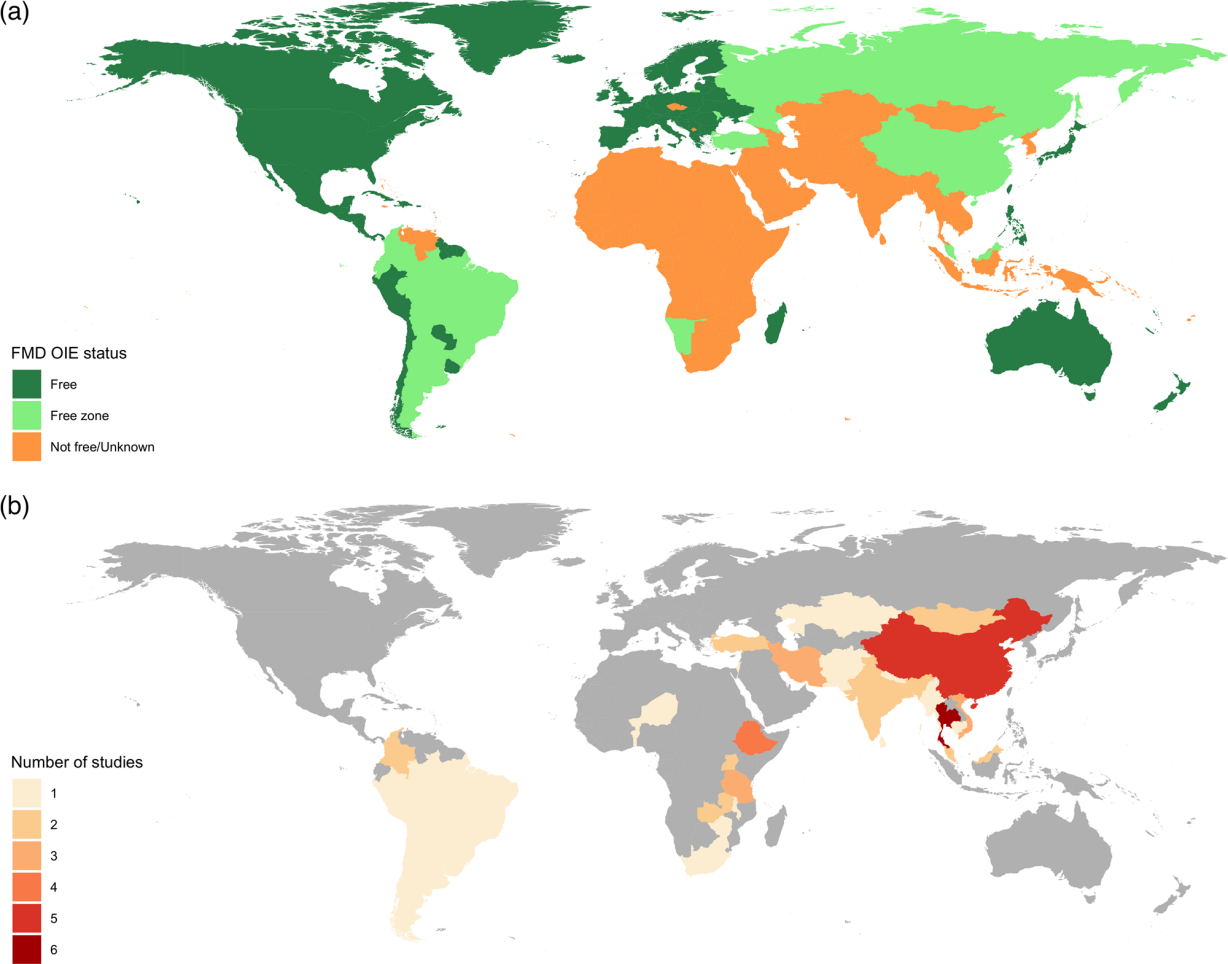
(a) Foot‐and‐mouth disease (FMD) situation based on World Organization for Animal Health (WOAH) foot‐and‐mouth disease status recognition. Regions in orange are endemic, report sporadic episodes or have an unregistered FMD status. (b) Geographical coverage of the studies included in this review. The map shows the number of studies included per country; countries in central and West, Central and North Africa were underrepresented, despite being endemic FMD regions.

FMD endemicity was defined based on the information available at the latest WOAH List of Members and Zones recognized as free from FMD (Resolution No 11, May 2022); this data was cross‐checked with the conjectured country status published by the FMD World Reference Laboratory (WRLFMD) to decide on the eligibility of a study based on the country in which it was conducted (World Organization for Animal Health, 2022b; World Reference Laboratory for Foot‐and‐Mouth Disease, 2022). Countries recognized as FMD free with or without vaccination were excluded from the analysis. However, studies conducted in countries certified as FMD free or with recognized free zones were eligible for inclusion if they were catalogued as endemic by the WRLFMD or if the study documented their historical path towards FMD freedom recognition (Figure 1). For reference, the WOAH defines an outbreak as the occurrence of one or more cases in an epidemiological unit (World Organization for Animal Health, 2021). A case refers to an individual animal infected by FMDv with or without clinical signs, whereas an epidemiological unit denotes a group of animals that share an epidemiological relationship making them likely to be at risk of exposure given their physical proximity or common husbandry practices. Hence, an epidemiological unit could either be a herd, a village or other of relevance in the context of counting FMD outbreaks. In this review, no formal FMD outbreak definition was adopted as a criterion for inclusion. All studies reported their own FMD case or outbreak definition, which were extracted for analytical purposes.

### 2.3 Information sources, literature search methods and study selection

A search strategy was designed and run in three electronic bibliographic databases (Embase, CAB Abstracts and Medline) through the Ovid platform (Table S2). MeSH and truncated free‐text terms were combined using Boolean and proximity operators to obtain a good balance between sensitivity and specificity while searching for the studies. Moreover, Google Scholar was used as the secondary source to retrieve relevant study reports. In this database, a general search strategy was designed and run; in addition, a search for each country that has not been certified by the WOAH as FMD free was included for validation purposes (World Organization for Animal Health, 2022b). Snowballing (citation screening) was used as a strategy to identify additional references from included studies and from published literature reviews that fell closely within the scope of this evidence synthesis (Premashthira et al., 2011; Souley Kouato et al., 2018).

**FIGURE 2:**
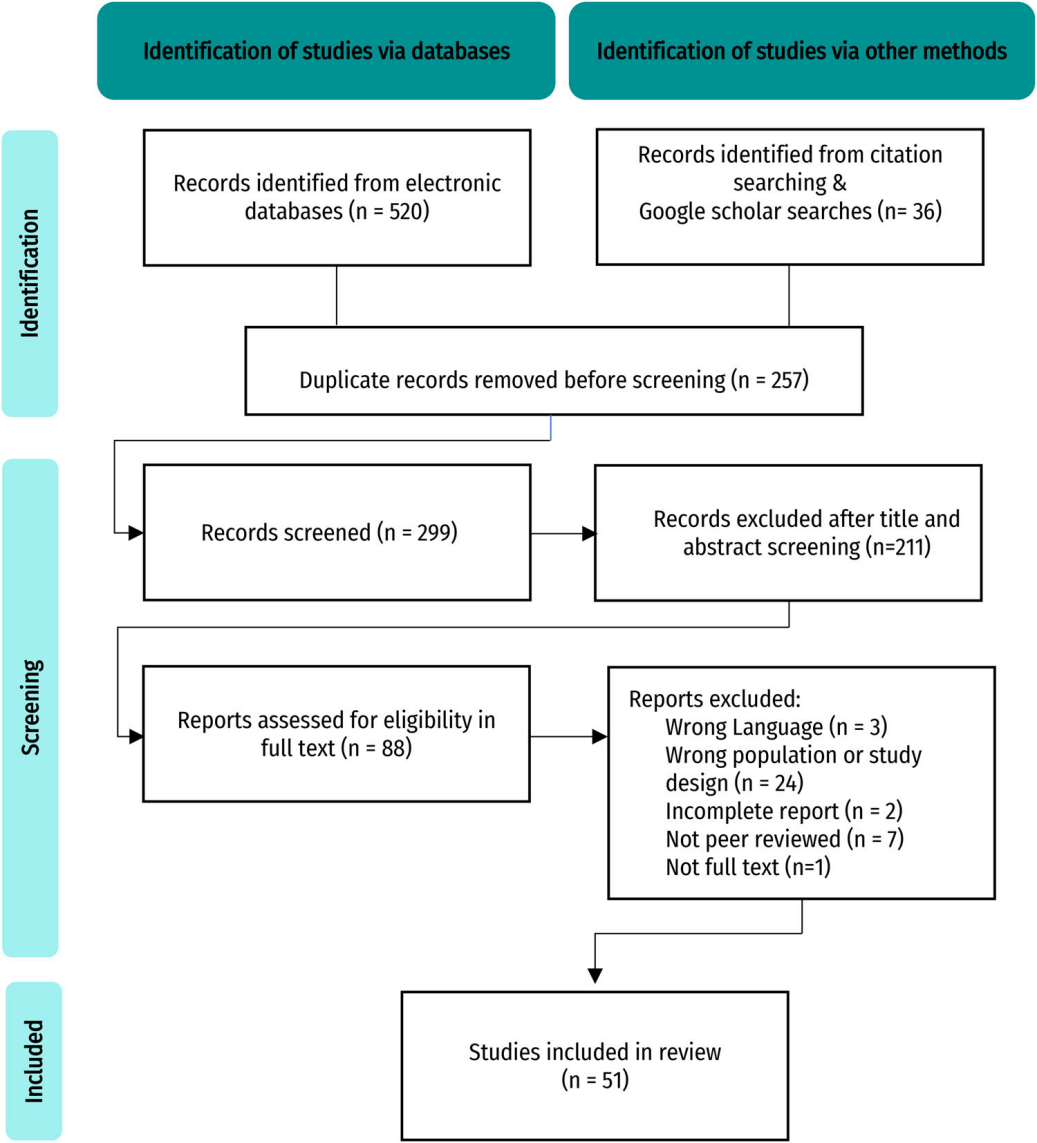
Flow diagram from bibliographic search of study records to final inclusion in the review

Electronic search outputs were imported to Mendeley. Duplicate studies were removed, and the title and abstracts were used for an initial screening of the reports. At this stage, irrelevant papers were excluded. Full texts were retrieved to assess the final inclusion based on the eligibility criteria. Definite inclusion was decided after reading the full texts by an individual reviewer. The reasons for exclusion after this stage were documented for traceability and transparency. Figure 2 shows a study of flow diagram.

### 2.4 Data extraction

Data from each study was extracted using a pre‐designed standardized form by an individual reviewer in a Microsoft Excel Spreadsheet which included: (a) study characteristics – author, year of publication, years analysed, country, geographical coverage, species and study aim; (b) FMD outbreak data and diagnostics – diagnostic criteria, outbreak definition, data source, surveillance system and serotypes; (c) data analysis and results – analytical tool type, clustering and pattern recognition method, temporal/seasonal assessment method, list of epidemiological factors studied and results.

### 2.5 Evidence synthesis and interpretation

**FIGURE 3:**
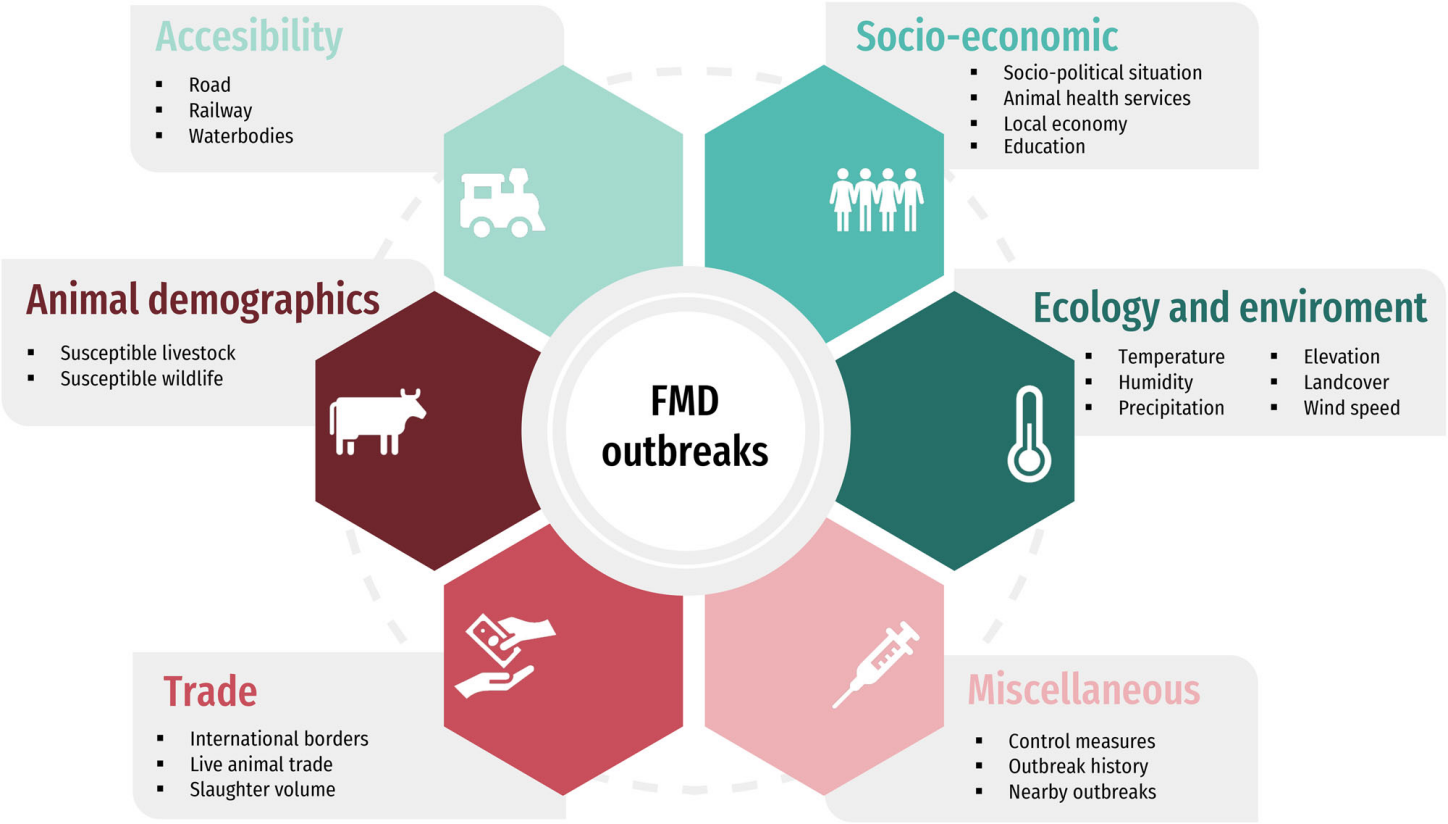
Conceptual framework used to classify the risk factors for foot‐and‐mouth disease outbreaks. A few examples of the epidemiological factors considered per category are included for reference.

The approaches to formally assess the presence of clusters were classified according to the dimensions and data forms proposed by Carpenter (2001) (Figure S3). A modified version of the framework proposed by Kanankege et al. (2020) was used as a guide to classify the analytical tools used for spatial or spatio‐temporal analysis based on their purpose as previously described. In addition, if a study used epidemiological factors for the purpose of risk analysis or to project the number or location of FMD outbreaks, these were identified and extracted. The factors examined in the epidemiological models commonly represented aspects with the potential to influence FMDv introduction, transmission, survival or features with a presumed role on the effectiveness of control measures. These factors were organized into a framework that included five main categories: (a) spatial accessibility; (b) animal demographics and livestock–wildlife interactions; (c) trade and commerce; (d) social and economic development; (e) ecology and environment. Epidemiological co‐variates that did not fit in any of these categories were grouped separately as ‘Miscellaneous’ (Figure 3; details on Table S4). All the information extracted from the studies was presented narratively, by synthesizing the core aspects connected to the review questions: study and outbreak characteristics, analytical methods and their results with an emphasis on the association between the epidemiological factors and the occurrence of FMD outbreaks. All analyses were conducted using R packages in RStudio version 4.0.4, which included *maps*, *ggplot2*, *webr* and summary functions within *dplyr* that were used to source maps, plot figures and to produce quantitative descriptions of the data (Becker et al., 2018; Hadley et al., 2022; Wickham, 2016).

## 3 RESULTS

### 3.1 Descriptive summary of included studies

A total of 51 studies were included through the combined electronic and manual search strategy after an initial pool of over 500 publications that were screened; 88 reports were assessed in full text and of those, 37 were excluded because they did not fulfil the eligibility criteria (Figure 2). Among the included studies, three documented national or regional paths towards FMDv control and WOAH recognition as an FMD‐free territory based on the analysis of retrospective case series of FMD outbreaks (Abdrakhmanov et al., 2018; Gallego et al., 2007; Sanchez‐Vazquez et al., 2019). All the characteristics of included studies are summarized in Table S5. The studies covered 39 countries distributed across three continents (Asia, Africa and America). The largest numbers of studies identified were from Thailand (*n* = 6), China (*n* = 5), Ethiopia (*n* = 4), Iran (*n* = 3), Tanzania (*n* = 3) and Vietnam (*n* = 3) (Figure 1). Most studies were conducted at a national scale using country‐wide FMD outbreak data (78.4%) available through official sources (92.1%). Frequently, the data were collected as part of passive surveillance systems (56.8%) in which FMD outbreaks were commonly diagnosed based on clinical presentation only (35.2%). However, the case and outbreak definitions, the spatial unit used for analysis and the livestock species for which outbreaks were recorded varied across studies (Tables S5 and S6). Some studies (33.3%, *n* = 17) included outbreak reports from more than one species, however, 31.3% of studies analysed FMD outbreaks recorded exclusively among cattle. The definition of FMD outbreak/case was included in 28 studies (54.9%), with the majority (*n* = 18, 64.2%) adopting the outbreak definition proposed by the WOAH or a modified version of it to account for a close temporal or spatial proximity in disease reports, the possibility of primary versus secondary outbreaks or to allow a broader spatial aggregation level (e.g. village, district and province).

In some studies, long‐term outbreak data was analysed; the longest being an 85‐year FMD outbreak case series from Zimbabwe (Guerrini et al., 2019). However, the study period varied across studies (median = 7 years; range: 1–85) with 14 studies analysing outbreak data collected for more than 10 years. A combination of analytical tools and purposes was identified in most reports, with studies mostly focusing on visualization and description of outbreaks (Figure S7).

### 3.2 Spatio‐temporal dependencies and pattern recognition

Almost half of the studies (49.0%, *n* = 25) used at least one spatial clustering analysis technique testing the non‐randomness hypothesis of spatial or spatial‐temporal distribution of outbreaks; of these, 52.0% (*n* = 13) and 68.0% (*n* = 17) used either a spatial or a spatial‐temporal tool, respectively. Five studies reported the use of several approaches for the identification of spatial or spatial‐temporal clusters in parallel. Point spatial clusters detected through Kulldorff's scan spatial statistic (*n* = 4), followed by Getis‐Ord (*n* = 2) and Cuzick‐Edwards (*n* = 2) were frequent, whereas Moran's I was often applied as a test for areal spatial clustering (*n* = 5). Kulldorff's scan statistic was chosen for simultaneous detection of space–time clusters (*n* = 17) in all studies whereas temporal and spatial autocorrelations were investigated through inverted correlograms (Gilbert et al., 2005). Spatio‐temporal directionality tests to detect the direction of the progression of FMD outbreaks were used in four studies (Alkhamis et al., 2009; Chen et al., 2020; Ma et al., 2017; Shiilegdamba et al., 2008).

Evidence of FMD spatial autocorrelation or the identification of disease clusters was documented in 80.0% (*n* = 20) of the studies. However, due to the diversity of techniques used to identify such clusters (unusual aggregation of outbreaks) and hotspots (excess level of outbreaks in comparison to a threshold level), their diverse assumptions and the configuration of the data, these results tended to vary within and across studies. Four studies reported heterogeneous results, documenting the identification of random or clustered patterns that varied across the periods, serotype or analytical method. For instance, Branscum et al. (2008) reported that annual variation influenced the identification of spatial association. Chen et al. (2020) and Ma et al. (2017) detected both clustering tendencies and random spatial patterns which varied across the years, serotypes and the various clustering methods used for analysis. Gilbert et al. (2005) reported a significant spatial autocorrelation structure for serotypes A and O, but not for serotype Asia‐1. The same study reported a strong temporal autocorrelation structure for serotype O with no such evidence for serotype A (Gilbert et al., 2005). The study of Hamoonga et al. (2014) was the only one that did not find any spatial dependence when analysing FMD outbreaks in Zambia. The methods used for cluster evaluation in the reviewed studies and their general findings are summarized in Table S8. Cluster size, measured through the radius of the significant clusters, and their duration greatly varied within and across studies which might be reflecting both the variations in the disease at the local level but also the differences in the tools used, hypothesis tested and the assumptions adopted.

### 3.3 Assessment of temporal and seasonal trends

Most studies (78.4%, *n* = 40) described the temporal trends of the outbreaks aggregated per day, week, month or year depending on the temporal aggregation of the original data. Conventional GLM or GLMM Poisson (Arjkumpa et al., 2020; Kerfua et al., 2018), negative binomial (Gunasekera et al., 2017), linear (Aman et al., 2020; Gallego et al., 2007; Jemberu et al., 2016; Perez et al., 2011; Woldemariyam et al., 2022) or logistic regression (Jemberu et al., 2016) models were used to explore or test hypothesis related to temporal trends. Other studies resorted to Bayesian approaches (Branscum et al., 2008; Choi et al., 2012; Gunasekera et al., 2022; Richards et al., 2014), additive models (Aman et al., 2020), spectral analysis (Perez et al., 2011), locally weighted regression (Sanchez‐Vazquez et al., 2019), normalized temporal trends (Madin, 2011), time series (Gallego et al., 2007), regression tree models (Souley Kouato et al., 2018) and models fitted to inverted correlograms (Gilbert et al., 2005) to analyse temporal data. Moreover, 13 studies formally analysed FMD seasonality through the calculation of seasonal indexes (Abdrakhmanov et al., 2018; Gallego et al., 2007; Perez et al., 2011), seasonal decomposition (Madin, 2011; Woldemariyam et al., 2022), randomness tests (Aman et al., 2020; Gallego et al., 2007; Jemberu et al., 2016) or by fitting frequentist or Bayesian models to temporal data (Choi et al., 2012; Guerrini et al., 2019; Jafarzadeh et al., 2014; Kerfua et al., 2018; Rahman et al., 2020). Punyapornwithaya et al. (2022) fit several time series methods, including ‘Seasonal Autoregressive Integrated Moving Average’ (SARIMA), ‘Trigonometric Exponential Smoothing State‐space mode with Box‐Cox transformation, ARMA errors and Trend and Seasonal components’ (TBATS), ‘Error Trend Seasonality’ (ETS), ‘Neural Network Autoregression’ (NNAR) and hybrid models to analyse the seasonal trends of outbreak episodes in Thailand.

Although their results are heterogenous, these studies often suggested that the temporal distribution of FMD outbreaks is not random and rather appears to display either cyclical fluctuations or seasonal patterns. In both temperate and tropical climates, studies in Asia, Africa and Latin America documented seasonal or temporal outbreak peaks that coincided with their respective dry/summer (Aman et al., 2020; Ayebazibwe et al., 2010; Dukpa et al., 2011; Jafarzadeh et al., 2014; Kerfua et al., 2018; Lee et al., 2020; Woldemariyam et al., 2022) or rainy/winter seasons (Choi et al., 2012; Hegde et al., 2014; Mondal & Yamage, 2014; Punyapornwithaya et al., 2022; Rahman et al., 2020; Ramanoon & Robertson, 2013). Other analysis reported multiple outbreak peaks across time series. These peaks encompassed combinations between the local dry and rainy seasons for different years, serotypes or locations (Abdrakhmanov et al., 2018; Gallego et al., 2007; Guerrini et al., 2019; Madin, 2011; Noudeke et al., 2017; Souley Kouato et al., 2018). In contrast, two studies reported the absence of a seasonal pattern (Jemberu et al., 2016; Perez et al., 2011). A common seasonal pattern for FMD outbreaks could not be identified, and the temporal distribution of outbreak based on their identification or reporting tended to vary across and within countries.

### 3.4 Modelling approaches

In addition to describing FMD outbreak distribution and patterns and identifying disease hotspots through clustering techniques, different spatial and spatial‐temporal tools were used to evaluate the association of several epidemiological factors on FMD outbreak identification and reporting. Of the studies included in this review, 23 (46.9%) used co‐variables to explore the link between population‐level epidemiological aspects and the outbreaks, to project FMD risk or to produce risk probability maps. Seventeen studies used epidemiological factors to model or predict FMD counts (47.1%, *n* = 8) (Branscum et al., 2008; Chhetri et al., 2010; Choi et al., 2012; Guerrini et al., 2019; Gunasekera et al., 2017; Hamoonga et al., 2014; Jafarzadeh et al., 2014; Kerfua et al., 2018) or to identify (or project) FMD risk areas (52.9%, *n* = 9) (Allepuz et al., 2015; Chimera et al., 2022; Gao & Ma, 2021; Gilbert et al., 2005; Gunasekera et al., 2022; Jemberu et al., 2016; Rahman et al., 2020; Sansamur et al., 2020; Souley Kouato et al., 2018). Two of these studies also addressed factors connected to disease notification (Chhetri et al., 2010; Jafarzadeh et al., 2014). Four studies focused only on forecasting FMD outbreaks using epidemiological factors as co‐variates to predict areal FMDv suitability, estimate spatial risk (number of outbreaks) or to detect spatial abnormalities in FMD passive surveillance data (Haoran et al., 2021; Perez et al., 2006; Richards et al., 2014; Sangrat et al., 2020).

A variety of analytical approaches were used. Conventional GLM and GLMM models were commonly reported, including Poisson (Guerrini et al., 2019; Kerfua et al., 2018), logistic (Chimera et al., 2022; Gilbert et al., 2005; Jemberu et al., 2016; Sansamur et al., 2020), zero‐inflated binomial (Jafarzadeh et al., 2014), negative binomial (Gunasekera et al., 2017) and geographically weighted regressions (Rahman et al., 2020). Six models implemented a Bayesian framework to model the spatial or spatial‐temporal distribution of FMD (Allepuz et al., 2015; Branscum et al., 2008; Chhetri et al., 2010; Choi et al., 2012; Gunasekera et al., 2022; Richards et al., 2014). Other spatial analysis included the use of co‐kriging method to assess the probability of FMD incidence (Perez et al., 2006); Maximum Entropy Ecological Niche (MaxEnt) modelling to detect suitable areas for FMDV serotypes (Gao & Ma, 2021); GIS‐based multi‐criterion decision analysis to predict suitability for FMD occurrence (Haoran et al., 2021; Sangrat et al., 2020); point process model to explain outbreak intensity (Hamoonga et al., 2014) and regression tree analysis to identify predictors and interactions that influence FMD occurrence (Souley Kouato et al., 2018).

### 3.5 Epidemiological factors linked to FMD outbreaks

**FIGURE 4:**
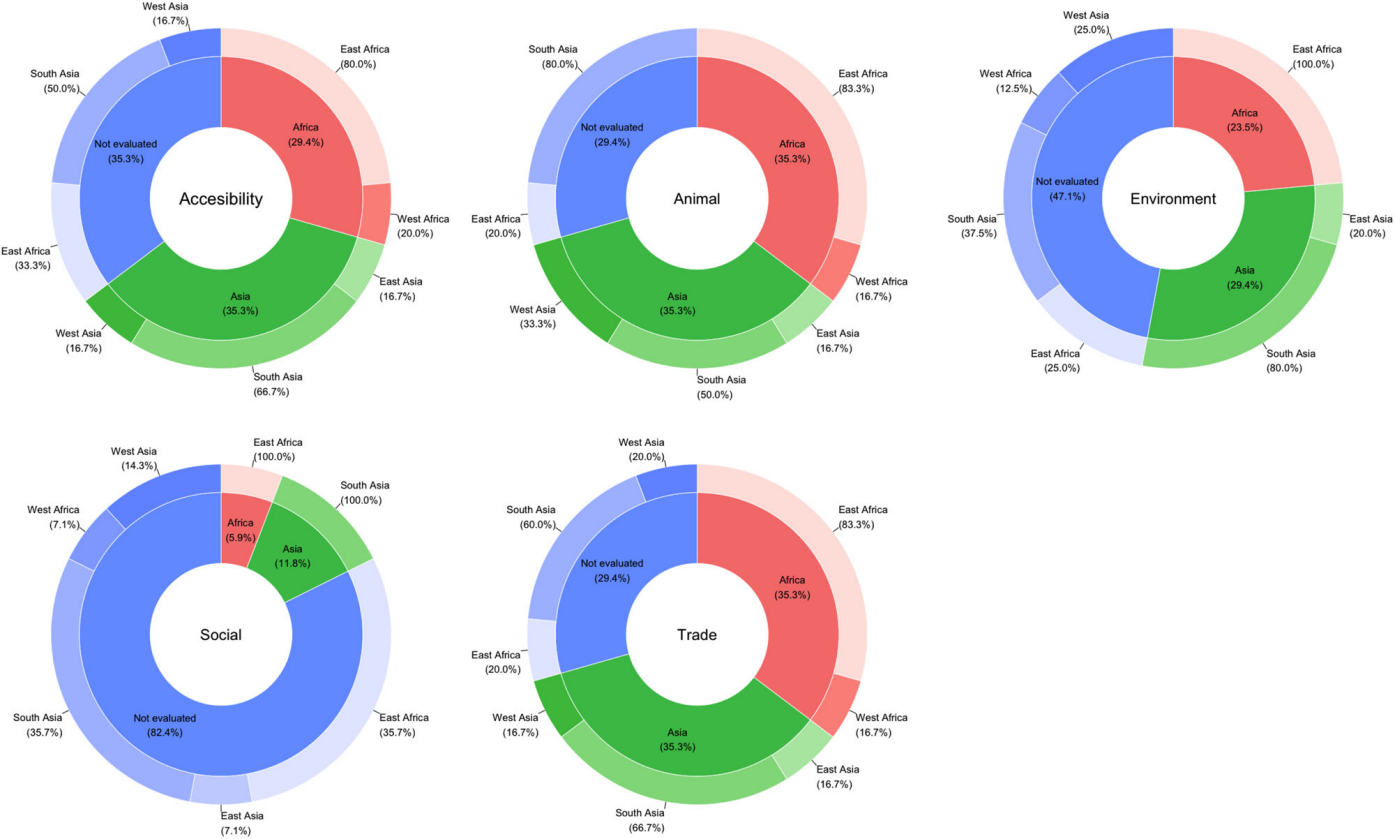
Proportion of models that included variables belonging to each category within the framework by UN region and sub‐region

**FIGURE 5:**
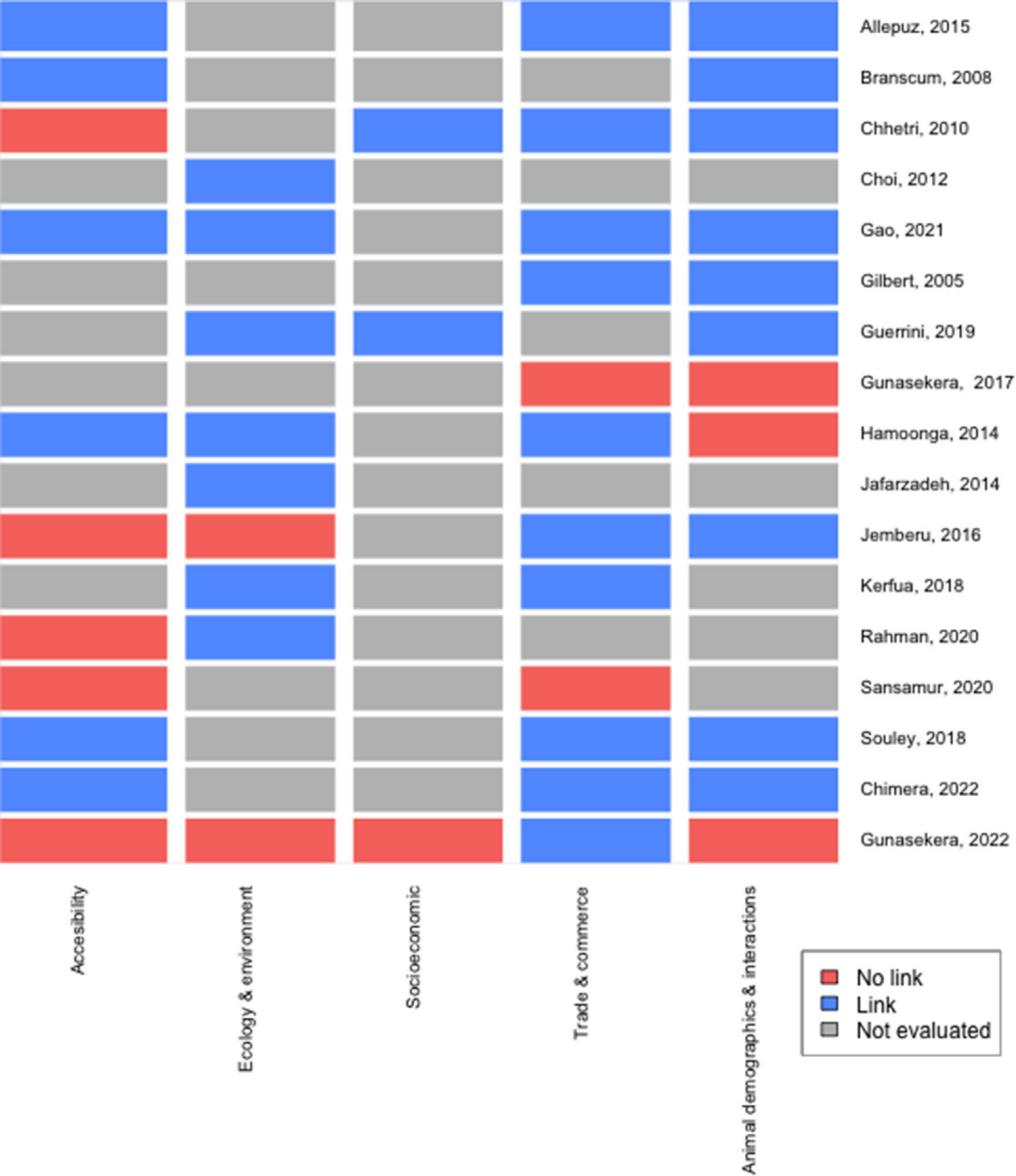
Categories of co‐variates used in the spatial or spatial‐temporal models. A link between any variable that belongs to a framework within the category is represented in blue, whereas no link is depicted in light red.

Although the epidemiological factors included in the models fell more frequently under the categories of animal demographics and livestock–wildlife interactions (*n* = 12), trade and commerce (*n* = 12), spatial accessibility (*n* = 11) and environment and ecology (*n* = 9), questions related to the role of socio‐economic aspects on FMD risk were also addressed as reported by three studies. Figure 4 shows the proportion of models that included variables belonging to each category within the framework by UN region and sub‐region; all the models were conducted with data collected in Africa and Asia, mainly in countries located in Eastern Africa and Southern Asia. Most of the models analysed the combined effect of risk factors that belong to more than one category within the framework (Figure 5); the outcome for each risk factor category is labelled as linked when at least one variable in the group was positively or negatively associated with the risk of FMD outbreaks. Except for socio‐economic aspects (Africa: 1/7; Asia: 2/10), more studies in Africa than in Asia explored the effect of animal demographics and livestock–wildlife interactions (Africa: 6/7; Asia: 6/10), trade and commerce (Africa: 6/7; Asia: 6/10), accessibility (Africa: 5/7; Asia: 6/10) and environment and ecology (Africa: 4/7; Asia: 5/10) in their models, when counted as a proportion to the total number of studies conducted in the region (Figure S9). Moreover, across studies, epidemiological factors, reflecting a similar risk pathway connecting them to the outbreaks, were included using different formats. Details of all the co‐variables studied are presented in Table S4.

### 3.6 Animal demographics and livestock–wildlife interactions

Twelve studies, six in each continent, addressed questions regarding the role of susceptible livestock species on FMD risk by including them in the models as livestock densities or population sizes, primarily to identify high‐risk locations for FMD (Table S4; Figure S9). Although many species were considered, cattle populations were more commonly reported as a co‐variate in comparison to the demographics of other susceptible species (e.g. goats, sheep, water buffaloes and pigs) (Allepuz et al., 2015; Branscum et al., 2008; Chhetri et al., 2010; Chimera et al., 2022; Gao & Ma, 2021; Gilbert et al., 2005; Gunasekera et al., 2022; Gunasekera et al., 2017; Hamoonga et al., 2014; Jemberu et al., 2016; Souley Kouato et al., 2018). Cattle density (or cattle population size) was associated with an increased FMD risk in 5 of 11 studies (Allepuz et al., 2015; Branscum et al., 2008; Chimera et al., 2022; Gao & Ma, 2021; Souley Kouato et al., 2018), whereas the linkage between small ruminant population and the outbreaks varied across studies with half (4/8) of the studies reporting an association with the risk of FMD outbreaks (Gao & Ma, 2021; Gilbert et al., 2005; Jemberu et al., 2016; Souley Kouato et al., 2018). Less often, studies described an association between pig or buffalo populations and the outbreaks (3/5) (Chhetri et al., 2010; Chimera et al., 2022; Gao & Ma, 2021). An epidemiological link between FMDv in wildlife and disease outbreaks in livestock was examined through the inclusion of co‐variates that represent the circulation of FMDv in wildlife reservoirs such as Cape buffalos. For example, proximity to protected, national parks or forest coverage were included in six analyses (five in Africa) as a proxy of a potential livestock–wildlife interaction; of these studies, three reported a positive association between presence or closeness to a protected area and FMD outbreak occurrence; however, this relationship varied across study periods, locations and serotypes (Allepuz et al., 2015; Chimera et al., 2022; Guerrini et al., 2019; Jemberu et al., 2016). Overall, this category was associated with FMD risk in 75% (9/12) of studies conducted in Asia and Africa (Figure S9).

### 3.7 Trade and commerce

Trade‐ and commerce‐related factors were included in 12 models, 6 in each continent (Table S4; Figure S9). In Africa, trade and commerce was associated with FMD risk in all studies that evaluated variables within this category, whereas in Asia, four out of six studies reported an association (Figure S9). Examining the effect of international connexions on FMD risk through proxy variables that represented the likelihood of an international trade network or cross‐border movements was a common objective, with seven studies (five in Africa and two in Asia), incorporating either the distance or adjacency to an international frontier into their models. An increased distance to an international border reduced the risk of FMD as reported by five studies (Allepuz et al., 2015; Chimera et al., 2022; Gunasekera et al., 2022; Hamoonga et al., 2014; Kerfua et al., 2018). However, this association was not always uniform; one study in Tanzania described heterogeneous results across the years (Allepuz et al., 2015). Moreover, factors related to market dynamics, including movement of live animals or their products, were also considered. Market location was an important contributing factor for the identification of outbreaks in China, Ethiopia and Niger (Gao & Ma, 2021; Jemberu et al., 2016; Souley Kouato et al., 2018). A few studies evaluated the association of human demography (Chhetri et al., 2010), slaughter volume (Chhetri et al., 2010; Gunasekera et al., 2017), abattoir location (Arjkumpa et al., 2020) and animal (Chhetri et al., 2010) and meat trade ratios (Gilbert et al., 2005) with FMD outbreaks. Heterogeneous results were reported for livestock meat demand gap as described by Gilbert et al. (2005). Slaughter‐related factors were assessed in three studies, all conducted in Asia. Sansamur et al. (2020) did not find a significant association between the distance‐to‐cattle abattoirs in a 5‐km radius and the location of farms that reported FMD outbreaks in Thailand. Furthermore, the numbers of animals slaughtered were not significant factors reported in the final risk models from Nepal and Sri Lanka (Chhetri et al., 2010; Gunasekera et al., 2017). Lastly, large human populations were linked to a higher likelihood of FMD reporting as described by Chhetri et al. (2010).

### 3.8 Accessibility and networks

Eleven studies (five in Africa and six in Asia) explored variables that represented landscape and topographical features, including permanent transport networks (e.g. roads and railways) and water bodies (e.g. rivers) to study FMD risk (Table S4; Figure S9). Most studies evaluating the role of accessibility in African countries (4/5) reported an association between spatial accessibility and risk of FMD; in contrast, two studies (out of six) analysing this aspect found an association in Asia. An increased distance to roads or railways was associated with decreased FMD occurrence (Allepuz et al., 2015; Chimera et al., 2022; Hamoonga et al., 2014), with higher FMD risk in areas with dense road networks or located closer to main transport routes (Gao & Ma, 2021). A study from Niger reported that the density of animal contacts quantified through a composite index (water crossing points, livestock markets and pastoral enclaves) was one of the main predictors for FMD occurrence (Souley Kouato et al., 2018). In addition, the influence of natural landscape features, such as the extension or distance to inland waterbodies and rivers, was assessed in four studies; of these, only one study from Turkey reported the protective effect of bordering a body of water against FMD occurrence (Branscum et al., 2008).

### 3.9 Ecology and environment

Although 9 studies (4 in Africa and 5 in Asia) assessed the relationship between environmental and ecological features and FMD outbreaks (Figure S9), 14 different epidemiological risk factors were reported in the studies reviewed (Table S4). These co‐variates were included in different formats with heterogenous results. The most commonly analysed factor were seasons (Choi et al., 2012; Guerrini et al., 2019; Jafarzadeh et al., 2014; Kerfua et al., 2018), temperature and precipitation (Choi et al., 2012; Gao & Ma, 2021; Gunasekera et al., 2022; Rahman et al., 2020). Other environmental and ecological indicators used in the reviewed papers are described in Table S4. Season impacted the temporal occurrence of FMD outbreaks in all studies (4/4); however, conflicting results were obtained for weather and other climate‐related co‐variables. Two studies explored the association between elevation and FMD risk, with locations at lower altitudes at higher risk of FMD outbreaks (Hamoonga et al., 2014). In addition, a positive contribution of isothermality and UV‐B with FMDv serotype A suitability was observed by Gao et al. (2021), whereas no association or contribution to FMD outbreaks was reported for diurnal range, agroecology, solar radiation, wind speed and land cover (Gao & Ma, 2021; Gunasekera et al., 2022; Jemberu et al., 2016; Rahman et al., 2020). Overall, environmental and ecological features were often associated to the risk of FMD in studies conducted in Asia (4/5) and Africa (3/4); however, the variables chosen to explore the risk differed across studies showed the diverse objectives of the analyses.

### 3.10 Social and economic development and miscellaneous

Socio‐economic data was only included in three studies (Table S4; Figure S9). Guerrini et al. (2019) analysed the variation in the number of FMD outbreaks among empirical socio‐economic periods in Zimbabwe, reporting an increased frequency of outbreaks in periods of political and socio‐economic crisis. Chhetri et al. (2010) focused on literacy rate and the provision of veterinary services (e.g. number of veterinarians, technicians and animal health workers) and found an association between the number of technicians in a district and increased outbreak reports at the village level in Nepal. In contrast, Gunasekera et al. (2022) assessed the effect of providing veterinary services across Indian states on the spatio‐temporal dynamic of reported FMD outbreaks, but this aspect was not retained in the best fit model. Miscellaneous co‐variables included temporal and spatial aspects, production systems and use of FMD countermeasures such as vaccination and prior or nearby outbreak reports. Recent outbreak reports in the same area or a nearby location were frequently linked to an increased FMD risk (Choi et al., 2012; Gilbert et al., 2005; Jafarzadeh et al., 2014). Moreover, one of two studies evaluating the impact of vaccination reported a decreased risk of FMD outbreaks in areas participating in FMD vaccination programmes (Gunasekera et al., 2022, 2017).

## 4 DISCUSSION

In this review, we have synthesized some of the key methodological aspects and findings of studies that use a spatial, temporal or spatio‐temporal analytical framework to describe FMD patterns, estimate its risk or project outbreak occurrence in endemic settings. We also critically appraised and summarized the epidemiological factors that have been used for FMD risk mapping and prediction. The large number of studies retrieved in the last two decades is indicative of the interest to understand FMD in endemic countries for risk mitigation planning and eventual elimination of viral circulation. This is consistent with the progression reported by the PCP‐FMD control strategy which, to date, has been successfully implemented in 72 countries classified into control stages zero to three (FAO et al., 2018), including eight countries with officially endorsed control programmes (World Organization for Animal Health, 2022b). Data visualization and descriptive exploratory analysis using georeferenced or areal data were the most frequent analytical tool used and the starting point for many reports. Descriptive results are the foundation of the outbreak analytics pipeline; the hypotheses generated at this stage normally precede and guide the development of more complex analyses and models that seek to gain in‐depth knowledge on the aspects influencing temporal or spatial disease variation (Kanankege et al., 2020; Polonsky et al., 2019). The progression from descriptive to analytical was a key feature of several study reports included in this review.

### 4.1 Temporal influence

Many infectious diseases are known to display cyclical, seasonal or secular (long‐term and consistent) trends, and based on our results, the same seems to be true for FMD outbreaks. Although the outbreaks exhibited a highly seasonal behaviour; their epidemic calendar was heterogenous with incidence peaks that varied across geographic locations and countries. Seasonal variation on disease incidence arises from the combination of seasonally linked introduction, transmission and other processes shaping the dynamics of at‐risk populations and the likelihood of viral survival (Martinez, 2018; Mielke & Garabed, 2020). Environmental factors, host behaviour and interactions (e.g. transmission‐relevant events derived from host contact rates, animal migrations and movements) and exogenous biotic factors (e.g. host‐virus–host‐ecosystem interface) are some of the relevant drivers to consider (Martinez, 2018). For instance, transhumance, nomadism and religious festivals are good examples of seasonal, agropastoral and cultural practices resulting in livestock mobility in Africa and some regions in Asia (Choi et al., 2012; Jafarzadeh et al., 2014; Motta et al., 2018; Pomeroy et al., 2019; Turner & Schlecht, 2019). These aspects are likely explanations for the shifting temporal dynamics of the outbreaks, possibly connected to varying levels of livestock interactions that can ultimately drive FMDv epidemics (Aman et al., 2020; Bronsvoort et al., 2004; Kim et al., 2016; Pomeroy et al., 2019).

Apart from animal movements, FMDv environmental survival could also play a significant role. Environmental survival of FMDv reportedly varies across seasons due to an interaction between relative humidity (RH) and temperature (Mielke & Garabed, 2020). Furthermore, FMDv is known to survive at different rates on inanimate surfaces and vegetation/food sources as described in experimental settings and field studies (Colenutt et al., 2018, 2022; Mielke & Garabed, 2020). At day 50, FMDv survival approaches 0% at 16°C and 37.5% RH during the dry season compared to ∼90% at 16°C and 86% RH during the wet season (Mielke & Garabed, 2020). The right environment at periods of increased animal contacts within and between groups can create the optimal conditions for viral transmission triggering introduction or superspreading events which may coincide with periodically increased number of outbreaks observed in some endemic areas (Ekwem et al., 2021; VanderWaal et al., 2022). This interaction has special implications in countries with territories located in the tropics where FMD is more heavily distributed.

### 4.2 Spatial dependence

Similarly, spatial dependency of FMD outbreaks was recognized in almost all studies that examined this aspect, irrespective of the analytical method used for testing. These results are important because, as for seasonal and temporal trends, outbreak cluster and hotspot detection can offer clues to the hidden causes of the excess in disease incidence and the drivers contributing to its endemicity (Carpenter, 2001; Lawson, 2010). Besides the obvious advantage of locating high‐risk areas for targeted surveillance, these analyses have the potential to inform decisions on the geographical extension and duration of post‐outbreak movement and trade restrictions, reactive (or pre‐emptive) vaccination and biosecurity measures. In some cases, this could help balance disease control interests and the socio‐economic burden of such outbreak response strategies (Limon et al., 2020; Tildesley et al., 2019). However, our results also show that geographical overlapping of cluster locations across and within tools did not always occur. This can be explained by the differences in the calculations behind them, the hypothesis tested, the assumptions adopted or to true differences occurring across periods (Carpenter, 2001). Although the quality of the outputs (e.g. geographical accuracy) relies heavily on the quality of the data, and despite some shortcomings of pattern recognition techniques, the consistent clustered nature of FMD outbreaks in endemic countries is indicative of underlying epidemiological processes facilitating or sustaining viral circulation. These processes can be explored through a holistic approach that understands disease risks as an interaction between epidemiological factors. Recognizing spatial and temporal variation is of relevance to forecast when and where FMD outbreaks could happen. However, any temporal, seasonal or spatial fluctuation should be interpreted in the light of other disease determinants, the limitations and strengths of the analytical tool used and the possible biases introduced from an imperfect surveillance system, a common problem across endemic settings (Combelles et al., 2019).

### 4.3 Modelling approaches

Spatial and spatio‐temporal models designed to estimate or project FMD risk were specified differently across studies. Frequentist statistical regression models were commonly used. However, unless spatial dependence has been ruled out or properly accounted for, these types of models may produce wrong estimates of disease risk and could mislead the conclusions of the analysis by disregarding one of the crucial aspects of spatial epidemiology: the presumed dependence between neighbouring units on disease frequency (Pfeiffer, 2008). In contrast, some authors opted for Bayesian approaches to model FMD outbreak data with the advantage of measuring inference uncertainty, the possibility of incorporating spatial or temporal structures through random‐effect terms for each location and a broad set of modelling classes readily available to approach different research questions, data types and distributions (Aswi et al., 2019; Pfeiffer, 2008). Given the diverse configuration of studies and the risk models, the results of each study should be interpreted bearing in mind the strengths and limitations of the modelling approach selected. This was one of the main sources of variation identified across studies included in this review. Despite this, we have aimed to detect common elements based on the information reported in each study to inform future research based on the current state of knowledge.

### 4.4 Variation in causal pathways

The foundation of each FMD model was a causal pathway characterizing the relationship between the outbreaks and the presumed epidemiological factors used to model disease risk as a large interactive system that explores spatio‐temporal variation. Complex or ‘simple’ causal pathways, influenced by data availability, contextual knowledge and previously reported risk factors, were assessed to undercover at‐risk areas. Many risk factors for FMDv have been documented on knowledge‐driven initiatives aimed to identify risk areas, predict FMDv suitable territories or develop participatory qualitative or quantitative risk assessments to estimate the risk of incursion and spread in endemic and at‐risk countries (Haoran et al., 2021; Sangrat et al., 2020). For instance, Dos Santos et al. (2017) extensively described the pathways associated with FMD occurrence; these pathways were connected to specific risk factors further categorized as (a) processes associated with viral introduction; (b) the exposure of susceptible animals; (c) the establishment of the infection in naïve populations, and (d) a dissemination step, in which the disease spreads to subsequent groups of animals once it is established. A similar logic largely describes what the spatial‐temporal models for FMD risk included in this review investigated. These were a mix of biological, landscape and human‐mediated mechanisms to explore disease introduction, survival, spread and notification.

#### 4.4.1 Accessibility and networks

Spatial accessibility, described in the models through railroad networks and location of large water bodies, was one of the factors linked to FMD risk. Studies used accessibility as a proxy for animal and human movements, representing direct or indirect (fomite) routes of viral exposure and transmission. Overall, the risk of FMD outbreaks decreased in less accessible locations. However, none of the studies used animal or human movement data to directly test this hypothesis. High accessibility may have a role in disease expansion; at the same time, some landscape features (such as rivers) can have the opposite effect and act as barriers for infection which might partially explain the low outbreak incidence in less accessible locations. Both findings are reasonable from the epidemiological perspective and have been explored in previous research (Bessell et al., 2008; Rivas et al., 2006, 2004). Yet, the impact of geographical accessibility on disease notification through passive surveillance systems and its role on timely outbreak control were often overlooked. Research conducted in Low and Low/Middle Income Countries(LMICs) shows that decreased geographical accessibility can either result in a higher infectious disease burden or lower disease reporting, which in turn reduces the understanding of true disease patterns (Hierink et al., 2021). The link between spatial accessibility and reporting seems plausible in the context of FMD, especially in remote areas (Limon et al., 2020; MacPhillamy, Olmo, et al., 2022; MacPhillamy, Young, et al., 2022); therefore, strategies to discern or quantify true disease absence from lack of reporting should be implemented (Madin, 2011; Richards et al., 2014).

#### 4.4.2 Animal demographics and livestock–wildlife interactions

Animal demographics and livestock–wildlife interactions impact FMD risk by representing the magnitude of the presence of susceptible population and, to a lesser extent, the possibility of infected and susceptible animals to meet. The evidence from the models generally supported an increased risk of FMD in cattle‐dense locations, highlighting its importance for disease mapping and forecasting. In contrast, the association between the outbreaks and the population of other at‐risk domestic species was heterogeneous. However, the role of small ruminants, pigs and buffaloes in FMD outbreaks should not be yet disregarded considering the growing volume of evidence showing variable levels of disease circulation, viral genetic diversity and unique features linked to the pathogenesis and viral epidemiology of FMDv in these species (Di Nardo et al., 2021; Stenfeldt et al., 2016). For instance, well‐documented variation in the viral serotypes and strains has been reported on SAT serotypes, successfully maintained in the wild by the African buffalo (Maree et al., 2016). Moreover, interspecies transmission events have occurred and are supported by evidence from phylogenetic and phylogeographic analysis (Brito et al., 2016; Di Nardo et al., 2021). Outbreak risk attributed to presumed contact with wildlife was confirmed in some models, although further investigations are required.

#### 4.4.3 Trade and commerce

Discrepancies in the supply and demand of livestock and livestock products within and across countries are one of the key risk factors of transboundary animal infections such as FMD. From an economic perspective, this market unbalance is evened out by increased trade and local commerce dynamics (formal and informal) which in turn can create opportunities for disease incursion and transmission. As part of this review, many variables, connected with trade and commerce, were examined. FMD outbreaks were linked to areas in which livestock markets operate or that are close to human consumption centres as evidenced by a link to the location of abattoirs and high human density locations. A growing body of evidence of viral circulation across cattle markets, highly populated areas and slaughterhouses backs up this epidemiological linkage (Buckle et al., 2021; Colenutt et al., 2022; Di Nardo et al., 2021; Gunasekara et al., 2021; Munsey et al., 2021). Cross‐border trade was also represented and was often connected to a higher outbreak risk. As indicated by Di Nardo et al. (2021), sharing an international border is one of the main predictors to FMDv diffusion (Di Nardo et al., 2021), especially in countries with porous international frontiers. Increased animal mobility is the common aspect for the operation of these market chains, with a clear part on FMDv endemicity and dispersal. However, indirect transmission routes could also be involved. Based on our results, the role of market and trade dynamics on FMD outbreak risk and prediction tools should be further studied. However, accurate local data on market dynamics is required to develop precise risk and predictive models.

#### 4.4.4 Ecology and environment

Environmental and climatic variables have the potential to influence FMD risk by affecting viral stability, survival or transmission. For instance, the risk of airborne transmission, a long‐haul route for FMDv spread, varies between geographical regions because of differences in husbandry systems, proximity of susceptible and affected species, animal densities and the local climate (Brown et al., 2022). Alternatively, weather‐related variables may be informative of the increased likelihood of animal‐to‐animal contacts driven by extreme climatic events such as drought, a factor that triggers human‐mediated movements of livestock and natural migrations in wildlife. A neutral pH (7–7.5), temperatures of less than 20°C and RH over 55% are the optimal conditions for FMDv stability and result in the extended survival of viral particles in the environment (Mielke & Garabed, 2020). As a result, the virus is expected to survive longer during the wet season in comparison to the dry season, as moist and cool conditions are favourable and delay its desiccation (Mielke & Garabed, 2020). Areas with conditions closer to the ideal for FMDv may be more suitable for its maintenance and are, theoretically, at higher risk of outbreaks. Hence, climatic, and microclimatic variables such as temperature, humidity, precipitation and windspeed may represent different mechanisms linked to direct and possibly indirect transmission routes of FMDv.

The results from the outbreak studies considered here were diverse and few associations between ecology and environment and FMD risk were identified. Aside from the possibility of a true null association, this could have been linked to the granularity of outbreak data. Aggregated (areal) data was commonly used which makes ecological inference more challenging and bias‐prone, especially for climatic and environmental modelling in which spatial resolution plays an important role due to high levels of local variation. As a result, environmental variables are probably better suited to model point data, for example, georeferenced outbreaks located in farms, households, or urban centres or to model serotype suitability based on presumed viral ecological niches as reported by Gao and Ma (2021). However, some ecological variables collected through remote sensing or aggregate indexes could be of value. Agroecology and land coverage have been used to estimate the location of farms or could shed some light on possible wildlife–livestock interactions relevant for FMDv transmission; in the absence of other data, this approach has proven useful to inform spatio‐temporal epidemic models, including FMD, and merit further exploration in the field of outbreak analytics in endemic contexts (Tildesley & Ryan, 2012).

#### 4.4.5 Social and economic development

Socio‐economic factors were poorly represented in FMD outbreak models, despite a well‐known relationship between disadvantaged socio‐economic position and infectious diseases (Loi et al., 2019; Wijayanti et al., 2016). Social determinants of animal health should be accounted for in models designed to understand and project FMD outbreaks (Card et al., 2018). This evidence can complement the results from recent research which highlights that the socio‐economic everyday reality of FMD affected communities might be associated with increased FMD outbreaks (Guerrini et al., 2019), and that in many cases, the same communities face several challenges to comply with the restrictions imposed as a result of the identification of the outbreaks (Limon et al., 2020; MacPhillamy, Young, et al., 2022). Including socio‐economic aspects in FMD spatio‐temporal modelling would allow to examine their importance and, if there is a linkage, to generate ideas for an integral strategy for FMD control that is not solely relying on the traditional methods (e.g. movement restrictions, market closures and vaccination). Studies offering the first clues of the connection between socio‐economic development, social instability and transboundary animal diseases are available in the literature providing a path forward to identify factors that could be studied in future models (Loi et al., 2019; Lubroth et al., 2017; Wajid et al., 2020).

### 4.5 Relevance to FMD surveillance and control in endemic settings

Outbreak science is a critical component for FMD preparedness and response. In endemic settings, outbreak analyses are the base of epidemic intelligence programmes to identify disease hotspots and to improve the design, tailoring or implementation of timely evidence‐based surveillance and response strategies. From a broader perspective, the identification of risk factors for the outbreaks can support the planning of ‘custom‐made’ population‐level interventions targeting epidemiological factors that enable disease introduction, establishment and dissemination. For example, options for a better management and traceability of the flow of live animals and animal‐derived products, sustainable strengthening of outbreak surveillance systems and shrinking the access gap to veterinary services could help booster existing control strategies by tackling key aspects associated with outbreak hotspots or through rapid and more efficient outbreak identification. All these could be implemented and adapted according to the local needs. An evidence‐based toolkit comprised by similar actions may shift the focus of outbreak management from a ‘crisis‐reactive’ approach to a multifaceted problem‐solving activity appropriate for long term disease surveillance and control.

### 4.6 Strengths and limitations

Context‐specific modelling and good quality data are essential to develop optimal models capable of accurately representing the local reality. Our results indicate that data collection is not harmonized; multiple outbreak and case definitions and different levels of granularity in the original data were identified. Detection biases resulting from a poor performance of outbreak surveillance systems arise and can result in under or over‐estimation of epidemiological associations with implications for disease mapping. In addition, these issues limited the scope and complexity of the analyses proposed, added uncertainty to the results and restricted the comparability of analytical outputs within and across countries and regions. From a methodological perspective, spatial‐temporal analyses such as those included here are prone to what is known as ‘ecological fallacy’; the loss of information, due to spatial aggregation of data and co‐variables used for inference, might mislead the conclusions of spatio‐temporal models (Spatial Aggregation and the Ecological Fallacy, 2010). These variations and limitations should be better thought of as model caveats and should be considered when interpreting analytical outputs.

While this review documents the use of a transparent and reproducible methodological approach for quantitative evidence synthesis, only studies published in English and Spanish were included to make the review more feasible. The extent and impact of the language bias on the results of the review, if present, are unknown. Countries located in Western, Northern and Central Africa were clearly underrepresented despite being historically affected by FMDv. In these areas, the research seemed to be more focused in understanding farm or individual risk factors (and perceptions) associated with seropositivity, rather than exploring population‐based drivers of the outbreaks or their spatial‐temporal patterns.

## 5 CONCLUSIONS

We retrieved a large body of research analysing outbreak data from a spatio‐temporal perspective, and we have shown that understanding and mapping FMD risk requires a systemic view. A number of epidemiological factors are involved, which regularly interact in complex ways. Variations across countries and regions are possible, although further scientific evidence is required to improve the understanding of local disease incursion and spread and the reasons behind variations in space and time. The knowledge gap with respect to the role and importance of socio‐economic drivers of FMD risk remains. This and other eco‐epidemiological factors should be further explored using different spatio‐temporal tools towards an integral and proactive epidemic approach focusing on outbreak prevention rather than control. However, there is a need to strengthen and standardize outbreak surveillance systems for up‐to‐date, granular and reliable data for accurate disease mapping and forecasting.

Despite its limitations, the use of spatial‐temporal tools and statistical modelling based on areal or point outbreak data allowed us to capture the bigger epidemiological picture and, as such, produced valuable information for decision‐making in data‐scarce environments. Complex transmission‐dynamic models looking to investigate host–FMDv interactions or to understand how the system could respond to the implementation of alternative countermeasures (e.g. vaccination, culling, movement restrictions, and community engagement interventions) can follow once there is a general understanding of the system and of the factors that could play a significant epidemiological role on the outbreaks. Given the broad epidemiological evidence (e.g. molecular, mathematical and statistical modelling) available in endemic countries, additional overviews of scientific evidence should be conducted to support regional roadmaps for FMD control through the planning of coordinated actions tackling recurrent issues.

## AUTHOR CONTRIBUTIONS

Conceptualization – L. González Gordon; writing: original draft preparation – L. González Gordon; writing: review and editing – T. Porphyre, D. Muhanguzi, A. Muwonge, L. Boden, B. M. de C Bronsvoort; visualization – L. González Gordon; supervision – T. Porphyre, A. Muwonge, L. Boden, B. M. de C Bronsvoor; funding acquisition: L. González Gordon, T. Porphyre, A. Muwonge, L. Boden, B. M. de C Bronsvoort. All the authors have read and approved the published version of this manuscript.

## CONFLICT OF INTEREST

The authors declare that they have no conflict of interest.

## ETHICS STATEMENT

The authors confirm that the ethical policies of the journal, as noted on the journal's author guidelines page, have been adhered to. No ethical approval was required as this is a review article with no original research data.

## Supporting information

Supporting Information

## Data Availability

All data generated through the review process is presented within the manuscript and its supporting information files.
