# Supplementary material for: A scoping review of foot‐and‐mouth disease risk, based on spatial and spatio‐temporal analysis of outbreaks in endemic settings
Source: Transbound Emerg Dis. 2022 Dec 13;69(6):3198–215. doi: 10.1111/tbed.14769 (PMC10107783; doi:10.1111/tbed.14769)

Supplementary information

Table S1. Preferred Reporting Items for Systematic reviews and Meta-Analyses extension for Scoping Reviews (PRISMA-ScR) Checklist

Section	Item	Prisma-ScR checklist item	Reported on page #
**Title**			
**Title**	1	Identify the report as a scoping review.	1
**Abstract**			
**Structured summary**	2	Provide a structured summary that includes (as applicable): background, objectives, eligibility criteria, sources of evidence, charting methods, results, and conclusions that relate to the review questions and objectives.	1
**Introduction**			
**Rationale**	3	Describe the rationale for the review in the context of what is already known. Explain why the review questions/objectives lend themselves to a scoping review approach.	2-3
**Objectives**	4	Provide an explicit statement of the questions and objectives being addressed with reference to their key elements (e.g., population or participants, concepts, and context) or other relevant key elements used to conceptualize the review questions and/or objectives.	4
**Methods**			
**Protocol and registration**	5	Indicate whether a review protocol exists; state if and where it can be accessed (e.g., a Web address); and if available, provide registration information, including the registration number.	NA
**Eligibility criteria**	6	Specify characteristics of the sources of evidence used as eligibility criteria (e.g., years considered, language, and publication status), and provide a rationale.	4
**Information sources***	7	Describe all information sources in the search (e.g., databases with dates of coverage and contact with authors to identify additional sources), as well as the date the most recent search was executed.	5
**Search**	8	Present the full electronic search strategy for at least 1 database, including any limits used, such that it could be repeated.	5
**Selection of sources of evidence†**	9	State the process for selecting sources of evidence (i.e., screening and eligibility) included in the scoping review.	5
**Data charting process‡**	10	Describe the methods of charting data from the included sources of evidence (e.g., calibrated forms	5-6

		or forms that have been tested by the team before their use, and whether data charting was done independently or in duplicate) and any processes for obtaining and confirming data from investigators.	
**Data items**	11	List and define all variables for which data were sought and any assumptions and simplifications made.	5-6
**Critical appraisal of individual sources of evidence§**	12	If done, provide a rationale for conducting a critical appraisal of included sources of evidence; describe the methods used and how this information was used in any data synthesis (if appropriate).	NA
**Synthesis of results**	13	Describe the methods of handling and summarizing the data that were charted.	6
		**Results**	
**Selection of sources of evidence**	14	Give numbers of sources of evidence screened, assessed for eligibility, and included in the review, with reasons for exclusions at each stage, ideally using a flow diagram.	7
**Characteristics of sources of evidence**	15	For each source of evidence, present characteristics for which data were charted and provide the citations.	7
**Critical appraisal within sources of evidence**	16	If done, present data on critical appraisal of included sources of evidence (see item 12).	NA
**Results of individual sources of evidence**	17	For each included source of evidence, present the relevant data that were charted that relate to the review questions and objectives.	8-13
**Synthesis of results**	18	Summarize and/or present the charting results as they relate to the review questions and objectives.	8-13
		**Discussion**	
**Summary of evidence**	19	Summarize the main results (including an overview of concepts, themes, and types of evidence available), link to the review questions and objectives, and consider the relevance to key groups.	12
**Limitations**	20	Discuss the limitations of the scoping review process.	19
**Conclusions**	21	Provide a general interpretation of the results with respect to the review questions and objectives, as well as potential implications and/or next steps.	20
		**Funding**	
**Funding**	22	Describe sources of funding for the included sources of evidence, as well as sources of funding for the scoping review. Describe the role of the funders of the scoping review.	20

Table S2. Example of the search strategy

**Database**	Embase <1980 to 2021 Week 45>, CAB Abstracts <1973 to 2021 Week 46>, Ovid MEDLINE(R) and Epub Ahead of Print, In-Process, In-Data-Review & Other Non-Indexed Citations, Daily and Versions(R)	Google scholar
**Search strategy**	1 exp Foot-and-Mouth Disease/<br>2 Foot-and-Mouth adj5 Diseas$).tw.<br>3 FMDV.tw.<br>4 exp Spatio-temporal/<br>5 spatial.tw.<br>6 temporal.tw.<br>7 exp Livestock/<br>8 livestock.tw.<br>9 exp Cattle/<br>10 cattle.tw.<br>11 cow$.tw.<br>12 exp Swine/<br>13 swine$.tw.<br>14 pig$.tw.<br>15 exp Sheep/<br>16 sheep.tw.<br>17 exp Goats/<br>18 goat$.tw.<br>19 (small adj3 ruminant$).tw.<br>20 1 or 2 or 3<br>21 4 or 5 or 6<br>22 7 or 8 or 9 or 10 or 11 or 12 or 13 or 14 or 15 or 16 or 17 or 18 or 19<br>23 20 and 21 and 22	(FMD AND risk AND spatial AND outbreak) + country
**Date**	1946 to July 28, 2021	July 28th 2022
**Output**	520 reports	5 first pages reviewed per search

Figure S3. Dimensions and data forms for classification of cluster analysis modified from Carpenter (2001)

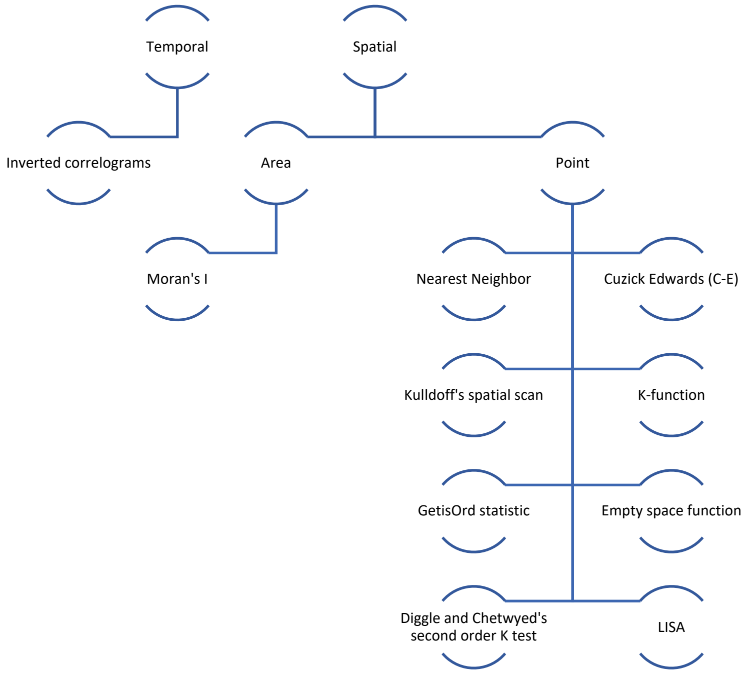

Table S4. Classification framework presenting the detailed list of epidemiological factors documented in the included studies

Epidemiological class	Scope	Co-variates	Reference
**Spatial accessibility**	Represents the connectedness of an area or the likelihood of a site being reached or entered from other locations.	Roads and/or railways – Distance to, density, presence, length, or distribution.	Allepuz, 2015; Chhetri, 2010; Chimera, 2022; Gao, 2021; Gunasekera, 2022; Hamoonga, 2014; Jemberu, 2016; Rahman, 2020; Sansamur, 2020.
		Water – Crossing points, border with body of water, length of rivers, distance to waterbodies or area/density of inland water.	Branscum, 2008; Chimera, 2022; Gunasekera, 2022; Rahman, 2020; Souley, 2018.
**Animal demographics and livestock-wildlife interactions**	Explores the multispecies nature of FMDv.	Livestock/Buffaloes population and/or density	Allepuz, 2015; Branscum, 2008; Chhetri, 2010; Chimera, 2022: Gao, 2021; Gilbert, 2005; Gunasekera, 2017; Gunasekera, 2022; Hamoonga, 2014; Jemberu, 2016; Souley, 2018.
		Distance to protected areas/national park or forest coverage	Allepuz, 2015; Chimera, 2022; Guerrini, 2019; Gunasekera, 2022; Hamoonga, 2014; Jemberu, 2016.
**Trade and commerce**	Examines market-related dynamics or aspects acting as a proxy for the likelihood of economic exchange of goods and services in a location.	International border – Distance to or adjacency	Allepuz, 2015; Chhetri, 2010; Chimera, 2022; Gunasekera, 2022; Hamoonga, 2014; Jemberu, 2016; Kerfua, 2018.
		Human population and/or density	Chhetri, 2010.
		Slaughtered volume and/or slaughterhouses presence/distance to	Chhetri, 2010; Gunasekera, 2017; Sansamur, 2020.
		Livestock markets – Presence or distance to	Chhetri, 2010; Gao, 2021; Jemberu, 2016; Souley, 2018.
		Animal exports/imports volume	Chhetri, 2010.
		Meat production/demand	Gilbert, 2005.
**Socio-economic development**	Tangible or intangible indicators of social, economic,	Political and economic situation	Guerrini, 2019.
		Veterinary services	Chhetri, 2010; Gunasekera, 2022.

	political, or cultural processes occurring with the capacity to influence the dynamics of FMDv.	Literacy rate	Chhetri, 2010.
		Urban population	Chhetri, 2010.
**Ecology and environment**	Landscape and climatic features studied in connexion to FMDv environmental circulation, stability, and survival.	Season	Choi, 2012; Guerrini, 2019; Jafarzadeh, 2014; Kerfua, 2018.
		Water availability and wetland areas	Guerrini, 2019; Hamoonga, 2014.
		Humidity	Choi, 2012.
		Temperature and precipitation	Choi, 2012; Gao, 2021; Gunasekera, 2022; Rahman, 2020.
		Diurnal range, isothermality and UV-B	Gao, 2021.
		Elevation	Hamoonga, 2014; Rahman, 2020.
		Agroecology	Jemberu, 2016.
		Solar radiation	Rahman, 2020
		Landcover	Rahman, 2020
		Wind speed	Rahman, 2020; 2022, Gunasekera, 2022
**Miscellaneous**	Any other aspect not represented in the other categories (e.g., transmission, control measures, production systems, etc)	Prior and or nearby outbreaks	Choi, 2012; Gilbert, 2005; Jafardezeh, 2014; Jemberu, 2016.
		Vaccination	Gunasekera, 2017: Gunasekera, 2022
		Time-Space	Gilbert, 2005; Gunasekera, 2017; Gunasekera, 2022; Kerfua, 2018; Souley, 2018.
		Production system or grazing area	Jemberu, 2016.

Table S5. Characteristics and description of included studies

Reference	Country	Period	Spatial coverage	Outbreak diagnostic criteria	Data source	Spatial analysis unit	Surveillance system	Serotypes
**Abdrakhmanov, 2018**	Republic of Kazakhstan	1955-2013	Country-wide	Laboratory confirmation	Official	Herd	Unclear	O, A
**Alkhamis, 2009**	Israel & Palestine	2006-2007	Local	Clinical	Official	Village	Unclear	O
**Allepuz, 2015**	Tanzania	2001-2006	Country-wide	Clinical	Official	Grid	Passive	NI
**Aman, 2020**	Ethiopia	1999-2016	Local	Clinical	Official	District	Passive	NI
**Arjkumpa, 2020**	Thailand	2008-2019	Country-wide	Clinical	Official	Farm	Passive	O, A
**Arjkumpa, 2020**	Thailand	2015-2016	Local	Mixed	Official	Farm	Unclear	NI
**Ayebazibwe, 2010**	Uganda	2001-2008	Country-wide	Unspecified	Official	Subcounty	Passive	NI
**Ayelet, 2012**	Ethiopia	2002-2006	Country-wide	Unspecified	Official	Region	Passive	NI
**Branscum, 2008**	Turkey	1996-2003	Country-wide	Clinical	Official	Province	Passive	NI
**Chen, 2020**	China	2010-2017	Country-wide	Unspecified	Official	Outbreak case	Unclear	NI
**Chhetri, 2010**	Nepal	2004	Country-wide	Unspecified	Official	District	Passive	NI
**Chimera, 2022**	Malawi	1957-2019	Country-wide	Laboratory confirmation	Official	Dip tanks	Passive/Active	O, A, SAT-1, SAT-2, SAT-3
**Choi, 2012**	Iran	1995-2002	Country-wide	Unspecified	Official	Province	Unclear	NI
**Dukpa, 2011**	Bhutan	1996-2008	Country-wide	Clinical	Official	Outbreak case	Passive	O, A, Asia-1, C

**Dukpa, 2011**	Bhutan	2007	Local	Unspecified	Official	Village	Passive/Active	O
**Gallego, 2007**	Colombia	1982-2003	Country-wide	Laboratory confirmation	Official	Town	Passive	O, A
**Gao, 2021**	China	2010-2019	Country-wide	Unspecified	Official	Outbreak case	Unclear	O, A
**Gilbert, 2005**	Turkey	1990-2002	Country-wide	Unspecified	Official	Province	Unclear	O, A, Asia-1
**Guerrini, 2019**	Zimbabwe	1931-2016	Country-wide	Unspecified	Official	Dip tanks	Unclear	NI
**Gunasekera, 2017**	Sri Lanka	2008-2014	Country-wide	Clinical	Official	Province	Passive	NI
**Gunasekera, 2022**	India	2008-2016	Country-wide	Laboratory confirmation	Official	State	Passive	O, A, Asia-1
**Hamoonga, 2014**	Zambia	1981-2012	Country-wide	Laboratory confirmation	Official	Ward	Unclear	O, SAT-1, SAT-2, SAT-3
**Hegde, 2014**	India	1977-2013	Local	Clinical	Official	Village	Passive/Active	NI
**Jafarzadeh, 2014**	Iran	1995-2002	Country-wide	Clinical	Official	Province	Passive	NI
**Jemberu, 2016**	Ethiopia	2007-2012	Country-wide	Clinical	Questionnaire	District	Active	O, A, SAT-1, SAT-2
**Kerfua, 2018**	Tanzania & Uganda	2011-2016	Local	Clinical	Official	Subcounty/Wards	Passive	NI
**Haoran, 2021**	China	2010-2020	Country-wide	Unspecified	Official	Outbreak case	Unclear	O, A
**Lee, 2020**	Vietnam	2007-2017	Country-wide	Clinical	Official	Commune	Passive	O, A
**Ma, 2017**	China	2010-2016	Country-wide	Unspecified	Official	Outbreak case	Unclear	O, A
**Madin, 2011**	South-East Asia	2000-2010	Continental	Clinical	Official	Outbreak case	Passive	O, A, Asia-1
**McFadden, 2015**	Mongolia	2010	Local	Mixed	Official	Herder	Passive/Active	NI

**Mondal, 2014**	Bangladesh	2010-2012	Country-wide	Clinical	Official	District	Passive	NI
**Noudeke, 2017**	Benin	2005-2014	Country-wide	Clinical	Official	Outbreak case	Unclear	NI
**Osmani, 2019**	Afghanistan	1995-2016	Country-wide	Clinical	Mixed	Province/District	Passive	O, A, Asia-1
**Pak, 2020**	South Korea	2014-2015	Country-wide	Unspecified	Official	Farm	Passive	NI
**Perez, 2005**	Iran	1996-2003	Country-wide	Unspecified	Official	Province	Passive	NI
**Perez, 2006**	Pakistan	1996-2000	Country-wide	Unspecified	Official	District	Passive	NI
**Perez, 2011**	China	2005-2009	Country-wide	Mixed	Official	Province	Passive	Asia 1
**Picado, 2011**	Tanzania	2001-2006	Country-wide	Clinical	Official	Village	Passive	NI
**Punyapornwithaya, 2022**	Thailand	2010-2020	Country-wide	Unspecified	Official	Area	Passive	NI
**Rahman, 2020**	Bangladesh	2014-2017	Country-wide	Mixed	Official	District	Passive	NI
**Ramanoon, 2013**	Malasya	2001-2007	Country-wide	Unspecified	Official	State	Passive	O, A
**Richards, 2014**	Vietnam	2006-2008	Country-wide	Unspecified	Official	Commune/Province	Unclear	O, A, Asia-1
**Sanchez-Vasquez, 2018**	South America	1968-2004	Continental	Unspecified	Official	First subnational administrative level	Unclear	C
**Sangrat, 2020**	Thailand	2014-2015	Country-wide	Unspecified	Unspecified	Subdistricts	Unclear	NI
**Sansamur, 2020**	Thailand	2017	Local	Mixed	Official	Farm	Passive/Active	NI
**Shiilegdamba, 2008**	Mongolia	2000-2002	Country-wide	Laboratory confirmation	Official	County	Passive	NI
**Sinkala, 2014**	Zambia	1981-2012	Country-wide	Laboratory confirmation	Mixed	Outbreak case	Passive	NI

**Sirdar, 2021**	South Africa	2005-2016	Local	Laboratory confirmation	Official	Dip tanks	Passive	SAT1, SAT2, SAT3
**Souley, 2018**	Niger	2007-2015	Country-wide	Clinical	Official	Region	Passive	NI
**Woldemariyam, 2022**	Ethiopia	2010-2019	Country-wide	Mixed	Official	District	Unclear	O, A, SAT-1, SAT-2

Table S6. FMD outbreak definition and species per study

Reference	Country	Species	Definition
Abdrakhmanov, 2018	Republic of Kazakhstan	Cattle, small ruminants & pigs	"An outbreak was defined as a single infected herd"
Alkhamis, 2009	Israel & Palestine	Not reported	"Outbreaks are defined according to the OIE as epidemiological Units (herds, farms, feedlots, or where premises could not be precisely delimited, areas with free grazing animals) in which at least one FMD-infected animal had been detected"
Allepuz, 2015	Tanzania	Cattle	"Households with FMD-affected stock were diagnosed by district veterinary officials on the basis of clinical signs"
Aman, 2020	Ethiopia	Cattle & small Ruminants	"An outbreak was defined as one of more cattle, sheep or goats showing FMD signs in a district"
Arjkumpa, 2020	Thailand	Cattle	"An outbreak is characterized as a cattle farm in which at least one animal displayed the typical clinical signs of FMD, including vesicles on the feet, mammary glands, and around the oral cavity"
Arjkumpa, 2020	Thailand	Cattle	"Outbreak farms were defined as cattle farms in which at least one animals was recorded with typical clinical signs of FMD, including vesicles on the feet, mammary glands, and around the oral cavity by district livestock officers, or tissue samples (e.g. oral epithelium clinical signs and vesicle lesion tissue) from animals with typical FMD clinical signs were confirmed as being FMDV positive by PCR method, or blood samples from such animals were positive to enzyme-linked immunosorbent assay technique"
Ayebazibwe, 2010	Uganda	Not reported	Not clear
Ayelet, 2012	Ethiopia	Cattle	Not clear
Branscum, 2008	Turkey	Not reported	OIE definition - "Occurrence of the disease in question in an agricultural establishment, breeding establishment or premises, including all buildings and adjoining premises, where animals are present"

Chen, 2020	China	Not reported	"A case is the number of animals suffering from FMD. The number of cases at the same geographical location is considered as an outbreak point, with the same outbreak ID in EMPRES"
Chhetri, 2010	Nepal	Not reported	Not clear
Chimera, 2022	Malawi	Cattle	"An outbreak was considered when one of more dip tanks (livestock inspection points) had confirmed FMD cases within the same month"
Choi, 2012	Iran	Not reported	Not clear
Dukpa, 2011	Bhutan	Cattle, small ruminants & pigs	"A case is defined as an animal with clinical signs or lesions characteristic of FMD with or without laboratory confirmatory diagnosis. An outbreak is defined as the occurrence of one of more cases of FMD in a herd, village, or sub-district. A case is considered a separate outbreak if it occurs in a herd or village separated from other herds or villages by physical barriers such as rivers, streams, hills, or mountains. Cases occurring at the same time in villages or herds that were contiguous were considered as one outbreak"
Dukpa, 2011	Bhutan	Cattle, small ruminants & pigs	"A village recording more than one outbreak within a 1-week period was considered to have only one outbreak because of the close proximity of herds and common management system"
Gallego, 2007	Colombia	Cattle & pigs	Not clear
Gao, 2021	China	Not reported	Not clear
Gilbert, 2005	Turkey	Not reported	Not clear
Guerrini, 2019	Zimbabwe	Cattle	"Primary outbreaks on the basis of their spatio-temporal distance (separated by time and/or locality) and within clusters, an outbreak was classified as primary if it was the first occurring within a detected cluster"
Gunasekera, 2017	Sri Lanka	Cattle & buffaloes	"An outbreak was considered as the reporting of several farms with clinically FMD infected cattle/buffalo from the nearby areas during the same time period"
Gunasekera, 2022	India	Cattle	Not clear
Hamoonga, 2014	Zambia	Cattle	"Laboratory confirmed diagnosis of FMD reported in a given ward, in a given year"

Hegde, 2014	India	Cattle	"An outbreak was defined as the occurrence of one or more clinical cases of FMD in a herd or village and each village was considered as an epidemiological unit for the purpose of defining and outbreak"
Jafarzadeh, 2014	Iran	Not reported	Not clear
Jemberu, 2016	Ethiopia	Not reported	"An FMD outbreak was defined by the occurrence of one or more cases of the disease in a district as clinically diagnosed by district animal health personnel. A continuous sequence of cases within a district was considered as one outbreak unless successive cases were separated by a time gap of at least a month"
Kerfua, 2018	Tanzania & Uganda	Cattle	"An FMD outbreak was defined as the presence of FMD clinical signs in at least one herd of cattle in a village within 1 month of the report of an outbreak"
Haoran, 2021	China	Cattle, small ruminants & pigs	Not clear
Lee, 2020	Vietnam	Cattle, small ruminants, pigs & buffaloes	Not clear
Ma, 2017	China	Cattle, small ruminants & pigs	Not clear
Madin, 2011	South-East Asia (Cambodia, Laos, Malasya, Myanmar, Singapore, Thailand, Vietnam)	Not reported	"A foot and mouth disease outbreak is the occurrence of FMD in one or more animals in a farm, or village, or group sharing a common area (e.g. pastureland, watering point, slaughterhouse, market, etc). All the cases occurring within 2 weeks of the previous case are considered as part of the same outbreak"
McFadden, 2015	Mongolia	Cattle small ruminants and camels	Not clear
Mondal, 2014	Bangladesh	Cattle, small ruminants & buffaloes	"A case is defined as an animal with a history, clinical signs or lesions characteristic of Anthrax, FMD, HS, PPR or dog bite/rabies"
Noudeke, 2017	Benin	Cattle	Not clear
Osmani, 2019	Afghanistan	Not reported	"A case was defined as an animal displaying clinical signs or lesions characteristic of FMD with or without confirmatory laboratory diagnosis. An outbreak was defined as the occurrence of one or more cases of FMD in a herd,

			village, district, or province within a defined time period (month/season) that was reported to the authorities"
Pak, 2020	South Korea	Not reported	Not clear
Perez, 2005	Iran	Not reported	"Outbreaks in the database are defined as "occurrence of the disease in question in an agricultural establishment, where animals are present"
Perez, 2006	Pakistan	Not reported	Not clear
Perez, 2011	China	Cattle, small ruminants & pigs	Not clear
Picado, 2011	Tanzania	Cattle	"An FMD outbreak was defined by the presence of one of more animals from a single cattle herd with clinical FMD"
Punyapornwithaya, 2022	Thailand	Not reported	"An outbreak episode of FMD was defined as an official report of an FMD outbreak in an outbreak area where disease investigation and FMD disease confirmation were undertaken by an authority from the Department of Livestock and Development (DLD)"
Rahman, 2020	Bangladesh	Cattle & buffaloes	Not clear
Ramanoon, 2013	Malasya	Cattle, small ruminants, pigs & buffaloes	Not clear
Richards, 2014	Vietnam	Not reported	Not clear
Sanchez-Vasquez, 2018	South America (Argentina, Bolivia, Brazil, Colombia, Chile, Paraguay, Peru, and Uruguay)	Not reported	Not clear
Sangrat, 2020	Thailand	Cattle, pigs & buffaloes	Not clear
Sansamur, 2020	Thailand	Cattle	"FMD outbreak farms were defined as dairy farms in which at least one animal was recorded with typical signs of FMD, including vesicles on the feet, mammary glands and around the oral cavity in 2016 by district livestock officers, or tissue sample from animals with typical FMD signs was conformed as being

			FMDV positive by applying ELISA at the Veterinary Research and Development Center Upper Northern Region, Lampang Province, Thailand"
Shiilegdamba, 2008	Mongolia	Cattle, small ruminants & camels	Not clear
Sinkala, 2014	Zambia	Cattle	"A case is defined as a clinical presentation of FMD-like lesions and confirmed by laboratory analysis"
Sirdar, 2021	South Africa	Cattle	"The unit of analysis (case) was defined as any dip tank where at least one domestic bovine showed FMD clinical signs"
Souley, 2018	Niger	Cattle	"A FMD outbreak was defined as the occurrence of one or more cases of the disease in a department as clinically diagnosed by veterinary officer"
Woldemariyam, 2022	Ethiopia	Cattle & small ruminants	“Two or more linked cases of the same illness or the situations where the observed number of cases exceed the expected number or where a single case of disease was caused by a significant pathogen in a specific period”

Figure S7. Analytical tool used and publication date of included studies

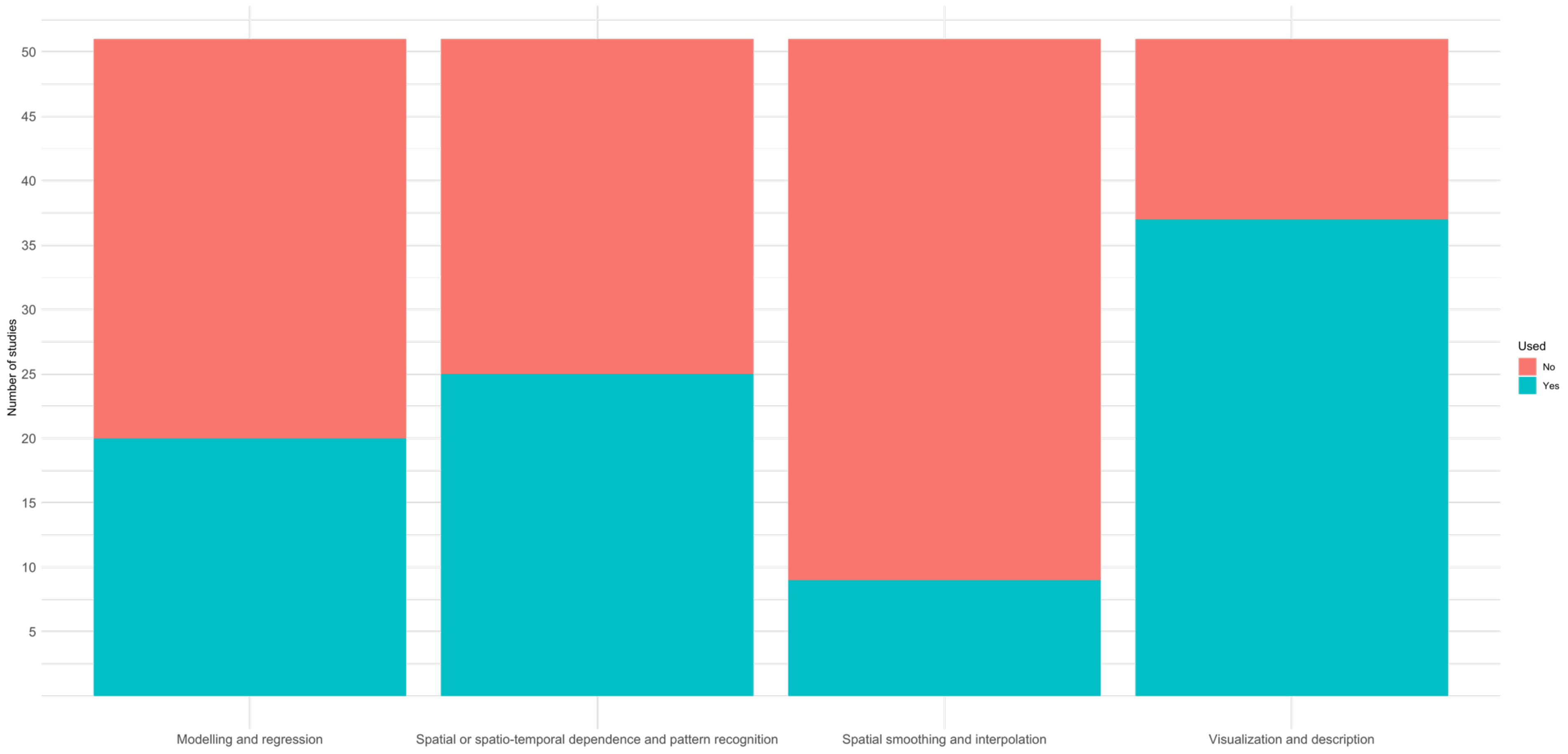

Table S8. Summary of methods investigating spatial, temporal or spatio-temporal clustering using FMD outbreak data

Reference	Time	Space	Time-space	Result	Details
Abdrakhmanov, 2018	NA	NA	Kulldorff's scan statistic	Clustered	Cluster radius (km): Serotype A: 71 ± 20; Serotype O: 82 ± 21<br><br>Cluster duration (days): Serotype A: 96 ± 45, Serotype O:116 ± 92
Alkhamis, 2009	NA	NA	Kulldorff's scan statistic	Clustered	Cluster radius (km): Range: 10.66 – 30.77<br><br>Cluster duration (days): Range 1 – 31
Allepuz, 2015	NA	Moran's I	NA	Clustered	NA
Arjkumpa, 2020	NA	NA	Kulldorff's scan statistic	Clustered	▪ STP:<br>Cluster radius (km): Range: 0.26 – 13.68<br><br>Cluster duration (days): Range: 6 – 46<br><br>▪ Poisson:<br>Cluster radius (km): Range: 0.66 – 42.95<br><br>Cluster duration (days): Range: 9 – 223<br><br>▪ Bernoulli:<br>Cluster radius (km): Range: 0.66 – 25.63<br>Cluster duration (days): Range: 28 – 119
Branscum, 2008	NA	Moran's I	NA	Heterogenous	NA
Chen, 2020	NA	Moran's I;<br>GetisOrd statistic	Kulldorff's scan statistic	Heterogenous	Cluster radius (km): Range: 121.49 – 1171.63<br><br>Cluster duration (days): Range: 4 – 684

Chhetri, 2010	NA	Kuldorff's scan statistic	NA	Clustered	Cluster radius (km): 23.27 – 26.31
Chimera, 2022	NA	NA	Kulldorff's scan statistic	Clustered	Cluster radius (km): Range: 33 – 50<br><br>Cluster duration (days): Range: 31 – 5475
Dukpa, 2011	NA	NA	Kulldorff's scan statistic	Clustered	Cluster radius (km): Range: 5.24 – 22.5<br><br>Cluster duration (days): Range: 30 – 245
Gao, 2021	NA	NA	Kulldorff's scan statistic	Clustered	Cluster radius (km): Serotype A: Range: 403.56 – 939.24; Serotype O: Range: 875.59 – 904.62<br><br>Cluster duration (days): Serotype A: Range: 9 –177; Serotype O: Range: 1 – 16
Gilbert, 2005	Inverted correlograms	Inverted correlograms	NA	Heterogenous	Short-distance spatial structure in 1997-2002 (159 and 190 km, respectively for serotype A and O)<br><br>Long-distance spatial structure between 1990-1996 (511 and 533 km, respectively for serotype A and O)
Guerrini, 2019	NA	Kuldorff's scan statistic	NA	Clustered	Cluster radius (km): 142.78 km
Hamoonga, 2014	NA	K-function	NA	Not clustered	NA
Lee, 2020	NA	NA	Kulldorff's scan statistic	Clustered	Cluster radius (km): Range: 43.95 – 176.69<br><br>Cluster duration (months): Range: 1 – 25
Ma, 2017	NA	Moran's I; GetisOrd statistic	NA	Heterogenous	NA
Madin, 2011	NA	Nearest neighbour distance function. (G); Empty space	Kulldorff's scan statistic	Clustered	Not reported

		function (F); Combination of G and J; Diggle and Chetwynd's second order K test			
Noudeke, 2017	NA	NA	Kulldorff's scan statistic	Clustered	Cluster radius: Not reported.<br><br>Cluster duration (days): Range: 61 –1767
Pak, 2020	NA	NA	Spatiotemporal score statistic (SC); Kulldorff's scan statistic	Clustered	Cluster radius (m): Range: 5,145 – 102,753<br><br>Cluster duration (days): Range: 30 – 63
Perez, 2005	NA	Kuldorff's scan statistic	NA	Clustered	Not reported
Picado, 2011	NA	NA	Kulldorff's scan statistic; K-function	Clustered	▪ k-function:<br>Cluster radius (km): Range: 20 – 300 km<br><br>Cluster duration (days): Range: 5 – 100.<br><br>▪ STP:<br>Cluster radius: Not reported<br><br>Cluster duration (days): Range: 21 – 133
Rahman, 2020	NA	Moran's I; GetisOrd statistic; Local indicators of spatial association (LISA)	Kulldorff's scan statistic	Clustered	Cluster radius (km): Range: 49.2 – 75.7<br><br>Cluster duration (days): Range: 91 –183

Shiilegdamba, 2008	NA	Cuzick-Edwards (C-E) test	Kulldorff’s scan statistic	Clustered	Not reported
Sinkala, 2014	NA	NA	Kuldorff's scan statistic	Clustered	Cluster radius (km): Range: 38.85 – 166.74<br><br>Cluster duration (years): 1 – 4
Sirdar, 2021	Kulldorff’s scan statistic	Cuzick-Edwards (C-E) test; Kulldorff’s scan statistic	Kulldorff’s scan statistic	Clustered	Cluster radius (km): Range 7.9 – 44.1<br><br>Cluster duration: Not reported
Woldemariyam, 2022	NA	NA	Kulldorff’s scan statistic	Clustered	Cluster radius (km): Range 26 – 380.9<br><br>Cluster duration (days): Range 1095 – 1460

Figure S9. Proportion of studies that report at least one co-variate linked to the risk of FMD outbreaks per risk class by sub-region. (A) Africa, (B) Asia

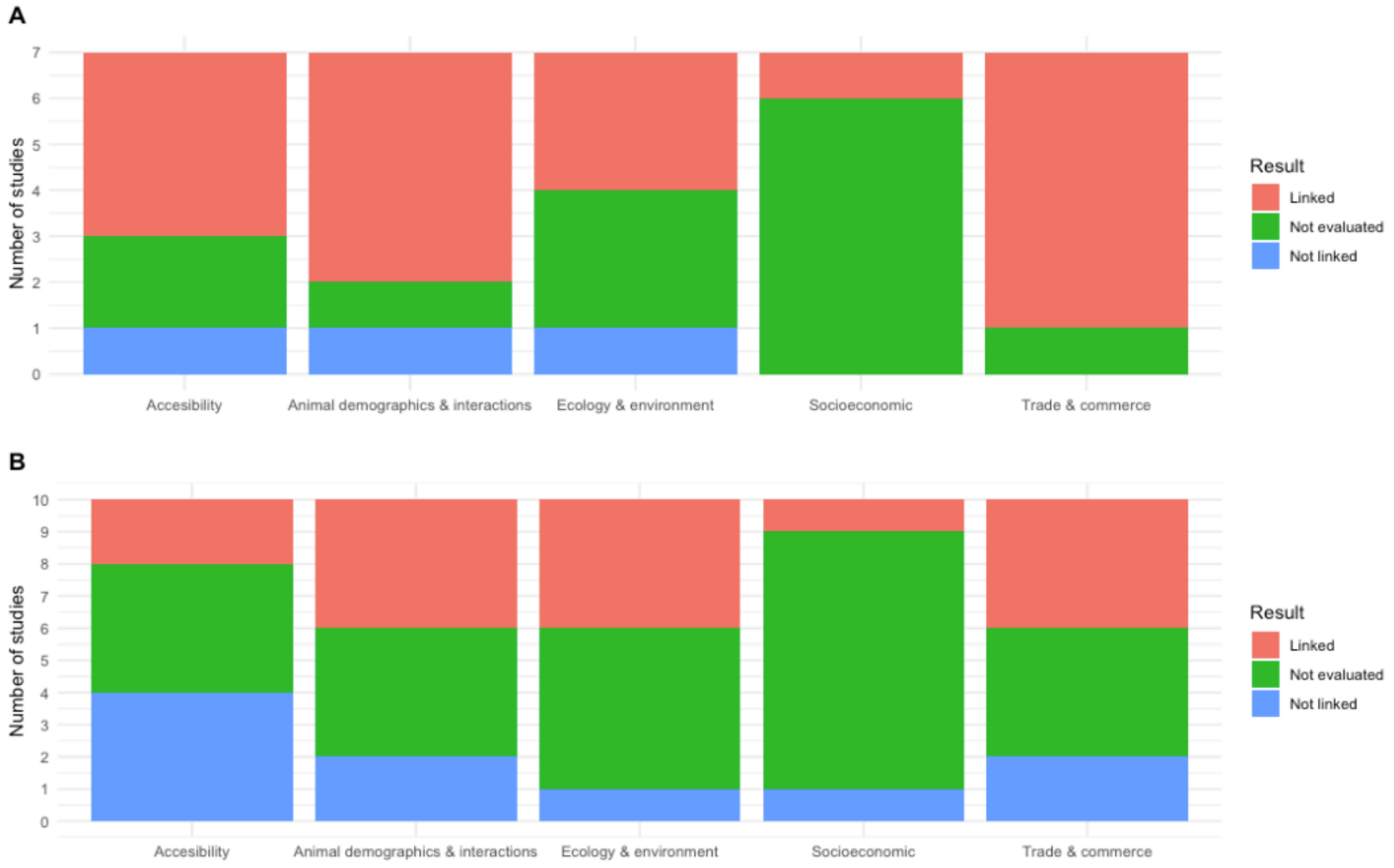

Supplement: Supplementary file 1 — Supporting Information [file TBED-69-3198-s001.pdf]
